# Myeloperoxidase Enzyme Activity in Feces Reflects Endoscopic Severity in Inflammatory Bowel Disease

**DOI:** 10.1093/ibd/izaf109

**Published:** 2025-05-24

**Authors:** Grace M Borichevsky, Akhilesh Swaminathan, Briana R Smith, Teagan S Edwards, Louisa V Ashby, Chris M A Frampton, Andrew S Day, Richard B Gearry, Anthony J Kettle

**Affiliations:** Mātai Hāora—Centre for Redox Biology and Medicine, Department of Pathology and Biomedical Science, University of Otago Christchurch, Ōtautahi Christchurch, AotearoaNew Zealand; Department of Medicine, University of Otago Christchurch, Christchurch, New Zealand; Department of Medicine, University of Otago Christchurch, Christchurch, New Zealand; Mātai Hāora—Centre for Redox Biology and Medicine, Department of Pathology and Biomedical Science, University of Otago Christchurch, Ōtautahi Christchurch, AotearoaNew Zealand; Department of Paediatrics, University of Otago Christchurch, Christchurch, New Zealand; Mātai Hāora—Centre for Redox Biology and Medicine, Department of Pathology and Biomedical Science, University of Otago Christchurch, Ōtautahi Christchurch, AotearoaNew Zealand; Department of Medicine, University of Otago Christchurch, Christchurch, New Zealand; Department of Paediatrics, University of Otago Christchurch, Christchurch, New Zealand; Department of Medicine, University of Otago Christchurch, Christchurch, New Zealand; Department of Gastroenterology, Christchurch Hospital, Christchurch, New Zealand; Mātai Hāora—Centre for Redox Biology and Medicine, Department of Pathology and Biomedical Science, University of Otago Christchurch, Ōtautahi Christchurch, AotearoaNew Zealand

**Keywords:** Crohn’s disease, ulcerative colitis, urine, glutathione sulfonamide

## Abstract

**Background:**

Concentrations of the neutrophil protein myeloperoxidase are elevated in the feces of individuals with endoscopically active inflammatory bowel disease (IBD). Its enzyme activity could give an immediate readout of endoscopic inflammation. We investigated whether fecal myeloperoxidase activity (fMPOa) is associated with IBD endoscopic inflammation. We also investigated whether myeloperoxidase promotes oxidative stress in IBD.

**Methods:**

Myeloperoxidase enzyme activity was measured using an enzyme-linked immunosorbent assay (ELISA fMPOa), a novel CM-sepharose extraction assay (CM-S fMPOa), or by quantifying urinary glutathione sulfonamide (GSA) by tandem mass spectrometry. GSA is a specific biomarker of myeloperoxidase activity. IBD activity was assessed using the ulcerative colitis endoscopic index of severity or the simple endoscopic score for Crohn’s disease (SES-CD). Spearman’s correlation and receiver operating characteristic curves evaluated biomarker utility.

**Results:**

IBD patients (*n* = 172) were recruited prospectively (ulcerative colitis, *n* = 72; Crohn’s disease, *n* = 100). fMPO was mostly active. Its enzyme activity, measured either as ELISA fMPOa or CM-S fMPOa, correlated with endoscopic inflammation in both ulcerative colitis and Crohn’s disease. Urinary GSA is also correlated with endoscopic disease inflammation. Correlations of urinary GSA with disease measures and other biomarkers were stronger in ulcerative colitis than in Crohn’s disease.

**Conclusions:**

Myeloperoxidase is active in IBD and its enzyme activity is a reliable marker of IBD endoscopic inflammation. Our results with the CM-S fMPOa assay demonstrate the potential for an immediate and accurate measure of fMPO enzyme activity as a robust, low-cost test for IBD activity. Myeloperoxidase may contribute to tissue damage in IBD.

## Graphical Abstract

**Graphical Abstract:**
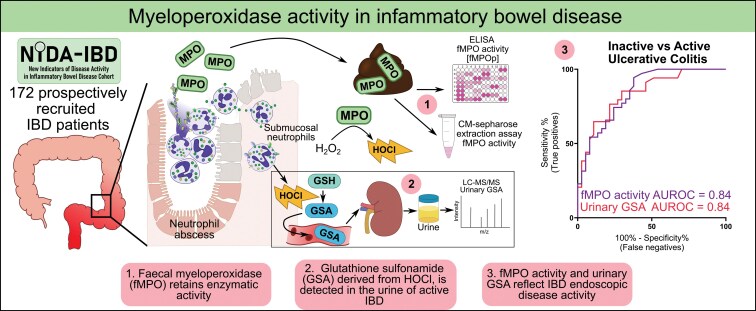

**Key Messages**

**What is already known?**

Fecal myeloperoxidase protein concentration is a biomarker of inflammatory bowel disease activity, but whether myeloperoxidase’s enzyme activity also reflects endoscopic inflammation has not been established.

**What is new here?**

Fecal myeloperoxidase enzyme activity, measured by an activity ELISA or after protein extraction, reflected endoscopic inflammation in IBD. A specific product of myeloperoxidase activity was elevated in urine from patients with more severe ulcerative colitis.

**How can this study help patient care?**

Our antibody-independent fecal myeloperoxidase extraction assay has the potential to be developed into an inexpensive and robust test that gives an immediate measure of IBD flares.

## Introduction

Neutrophils are a major immune cell type in the lesions of patients with inflammatory bowel disease (IBD). Their importance and presence in disease are reflected in the histological evaluation of ulcers and by the use of the neutrophil protein fecal calprotectin (fCal) as the currently accepted clinical biomarker of IBD activity.^1,2^ Previous studies, including our own, have shown that the abundant neutrophil enzyme myeloperoxidase is also elevated in the feces of individuals with endoscopically active ulcerative colitis or Crohn’s disease.^3^ One advantage of measuring myeloperoxidase over calprotectin is that its enzyme activity can be measured independently from its protein concentration.^4^ This enzyme activity could be exploited to give an immediate indicator of disease inflammation. However, there has been little work investigating fecal myeloperoxidase (fMPO) enzyme activity in IBD. In previous work using an ELISA that measures both MPO protein and MPO activity, we found that MPO activity strongly correlated with fMPO protein concentration in a small group of IBD patients.^3^ This work needs to be extended to a large cohort of patients so that fMPO activity can be related to disease severity and types. Also, the recovery of myeloperoxidase from feces, and potential difficulties in measuring it directly, need to be critically evaluated.^5^

A notable feature of myeloperoxidase is its relatively high isoelectric point of 11, which renders it positively charged under physiological conditions. Its inherent cationic nature makes myeloperoxidase sticky and difficult to measure in biological samples.^6^ However, its high isoelectric point can be manipulated to extract the protein from complex mixtures using cationic exchange media like CM-sepharose. This principle is commonly used for the purification of myeloperoxidase from neutrophils.^7^ Cation exchange could be used to purify fMPO and enable measurement of its enzyme activity without the requirement of antibody capture or detection. This approach could lower the cost and lengthen shelf stability compared to existing ELISA kits for fMPO. Thus, measurement of fMPO enzyme activity is potentially a cost-effective strategy to perform a noninvasive assessment of gut inflammation.

The major physiological product of myeloperoxidase’s enzyme activity is hypochlorous acid (HOCl), which if produced excessively could damage host tissue through oxidation of critical proteins. HOCl also reacts favorably with the physiological antioxidant glutathione. Concentrations of glutathione are lower in the intestinal mucosa of patients with IBD compared to healthy controls.^8–10^ HOCl reacts with glutathione to form glutathione disulfide and the HOCl-specific and irreversible product glutathione sulfonamide (GSA).^11–14^ We have previously detected elevated GSA in serum, bronchoalveolar lavage fluid, and urine of children with infectious complications of cystic fibrosis, showing that this marker is associated with periods of neutrophilic inflammation.^15^

In this study, we aimed to extend our previous work on fecal myeloperoxidase in a prospectively recruited cohort of patients with IBD who had endoscopic evaluation.^3^ Using this cohort of patients, we have now evaluated measures of fecal myeloperoxidase enzyme activity as noninvasive biomarkers of inflammation. fMPO activity was measured directly by ELISA (ELISA fMPOa), or by an antibody-independent extraction method (CM-S fMPOa) and correlated with validated indices of endoscopic and clinical IBD activity. Myeloperoxidase activity was also assessed by measuring urinary GSA using liquid chromatography-mass spectrometry (LC-MS/MS).^14,15^

## Materials and Methods

### Materials

A full list of materials used in this study are provided in the Supplementary Methods.

### Study Populations

The NIDA-IBD cohort comprised a repository of IBD patient samples with prospectively measured endoscopic and symptomatic disease activity. This study was approved by the New Zealand Health and Disability Ethics Committee (18/NTA/197) and was conducted in accordance with the World Medical Assembly Declaration of Helsinki. The details of participant eligibility, descriptive details of study participants, and information about the collection and storage of samples of the NIDA-IBD cohort have been previously described.^3^ A detailed description of biological sample collection is provided in the Supplementary Methods. In brief, all NIDA-IBD study participants provided biological samples (blood, urine, and stool) in addition to completing symptom questionnaires prior to endoscopic disease assessment.

Fecal samples of healthy controls were taken from the Christchurch irritable bowel syndrome (IBS) cohort to investigate mechanisms for gut relief and improved transit (COMFORT) cohort; this included patients with IBS or controls who had a colonoscopy performed to exclude malignancy or IBD.^16^ The COMFORT study protocol was reviewed by the Northern A ethics committee and granted approval (Ref16/NTA/21).

Healthy control urine samples were collected from 10 healthy individuals that reflected the age-sex-matched demographics of the NIDA-IBD cohort.^3^ Ethical approval was obtained from the Southern Health & Disability Ethics Committee, New Zealand, abiding by the Declaration of Helsinki principles.

All study participants gave written informed consent.

### Disease Activity Assessment

The disease status of participants’ IBD was assessed previously using validated scoring systems for clinical, symptomatic, and endoscopic aspects of their disease.^3^ The partial Mayo score and Simple Clinical Colitis Activity Index (SCCAI) were used to assess ulcerative colitis clinical activity.^17,18^ The Crohn’s Disease Activity Index (CDAI) and Harvey Bradshaw Index (HBI) were used to assess Crohn’s disease clinical activity.^19,20^ Symptom questionnaires were completed by study participants prior to their baseline ileocolonoscopy.

Endoscopic disease activity was assessed by the Ulcerative Colitis Endoscopic Index of Severity (UCEIS) and the Simple Endoscopic Score for Crohn’s Disease (SES-CD).^21,22^ Three individuals with ileal (*n* = 1) or ileo-colonic (*n* = 2) Crohn’s disease had their disease assessed by magnetic resonance imaging (MRI); all 3 were found to have active Crohn’s disease. Endoscopic activity was assessed by gastroenterologists trained in carrying out these assessments who were blinded to study biomarkers and independent of study investigators. Patients were grouped into inactive, mild, moderate, or severe endoscopic activity as defined by their endoscopic scores (Supplementary Table S1). Due to smaller numbers of patients with severe disease, moderate and severe groups were combined for analysis.

### CRP Measurement

We previously measured c-reactive protein (CRP) in blood samples from the NIDA-IBD cohort using automated methods at a commercial clinical laboratory, Canterbury Health Laboratories (Canterbury, New Zealand).^3^

### fCal Measurement

Previously, fecal samples were solubilized using the Calpro Easy Extract devices (Calpro AS, Norway). Fecal calprotectin (fCal) was then measured using the CALPROLAB ALP Calprotectin ELISA kit (Calpro AS, Norway), according to the manufacturer’s directions.^3^

### ELISA-Measured fMPO

The ELISA to measure fMPO enzyme activity (ELISA fMPOa) and protein concentration (ELISA fMPOp) was conducted as previously described.^3,23^ A summary of the fMPO ELISA protocol inclusive of measuring enzyme activity is included in the Supplementary Methods. In short, after the capture of myeloperoxidase by the monoclonal antibody, the enzyme activity was measured by oxidation of the peroxidase reagent AmplexUltraRed in the presence of hydrogen peroxide (H_2_O_2_). Next, the myeloperoxidase protein concentration was assessed as a sandwich ELISA via a phosphatase-derived colorimetric reaction.

The recovery, variability in whole stool, and stability of fMPO enzyme activity and specific activity were compared to fMPO protein concentration as previously described (Supplementary Figure S1–S3).^3^ The specific activity of fMPO was calculated as fMPO activity (μg/g)/fMPO protein concentration (μg/g)) × 100.

### CM-Sepharose fMPO Extraction Method

An in-depth protocol of the CM-sepharose extraction assay for fMPO activity (CM-S fMPOa) is provided in the Supplementary Methods. In brief, solubilized fecal samples were equilibrated with the cation exchange resin, CM-sepharose. The beads, with bound myeloperoxidase, were washed with a detergent-containing buffer, followed by acetonitrile to remove interfering compounds, then with buffer to rinse acetonitrile from the resin. Myeloperoxidase activity was measured using the peroxidase substrate AmplexUltraRed and H_2_O_2_. The reaction was stopped by the addition of 40 μg/mL catalase. The oxidized, pink fluorophore was eluted from the resin by acetonitrile and its fluorescence was measured using a plate reader. Purchased human myeloperoxidase purified from peripheral blood was processed in an identical manner alongside fecal samples, including extraction by CM-Sepharose to generate a standard curve. Samples with fMPO activity exceeding the upper limits of the standard curve were diluted in CTAB fecal solubilization buffer (Supplementary Materials) prior to extraction by CM-sepharose.

Recovery of myeloperoxidase from fecal samples was calculated by spiking solubilized fecal samples with known concentrations of myeloperoxidase, subtracting the signal from unspiked samples, and comparing the recovery to a spiked buffer equivalent.

### Inhibitor Experiments

Dapsone and AZM198 were used to evaluate the specificity of the CM-S fMPOa assay.^24^ These peroxidase inhibitors were added to the ELISA after the capture of myeloperoxidase by the primary antibody, or after the final buffer wash for the CM-sepharose extraction assay. Samples were incubated with either 10 μM H_2_O_2_ (H_2_O_2_ control), 50 μM dapsone/10 μM H_2_O_2_, or 5 μM AZM198/10 μM H_2_O_2_. This preincubation was for 20 min at room temperature (22 °C) before adding the specified assays’ myeloperoxidase enzyme reaction mixtures. Peroxidase activity of dapsone or AZM198 treated samples was expressed as a percentage of the measured fluorescence of the H_2_O_2_ control (100%).

### Urinary GSA

Urinary GSA was measured using LC-MS/MS with a stable isotype internal standard as previously described.^14,15^ The lower limit of quantification of GSA was 0.02 μM. Urinary GSA below this level was set at 0.019 μM. Urine specific gravity (SG), measured on a refractometer PAL-10S (ATAGO, Japan) standardized the concentration of urinary GSA to the osmolarity of urine samples. Standardized urine concentrations for analytes of interest were calculated using the equation below, with the value of 1.02 set as an “average” SG of human urine.^15^ Samples with a SG above or below the limit of detection on the refractometer (*n* = 2) were excluded from analysis. Standardized urinary GSA ranged from 0.0613 μM to 0.8901 μM.

## Equation


$$\begin{array}{l} {\left[Analyte\right]}_{corrected}={\left[Analyte\right]}_{measured}\times \;\frac{1.02-1}{SG-1} \end{array}$$

### Statistical Analysis

Spearman rank correlation coefficients were used to test the associations amongst the biomarkers and between biomarker levels and clinical measures. Kruskal–Wallis non-parametric analysis of variance (ANOVA) was used to compare the biomarker levels between IBD endoscopic activity and classification groups, with Dunne’s planned multiple comparisons subsequently performed. For subgroup analysis of isolated ileal Crohn’s disease (Montreal location classification L1) Mann Whitney *U*-test was performed to test for the difference between active and inactive ileal disease. These tests were performed on GraphPad Prism 10 (version 10.2.3 GraphPad Prism, Boston, USA).

#### Receiver operator characteristic curves

The degree of gut inflammation as assessed by endoscopic activity scores were classified in 2 ways: less severe (remission-mild) versus more severe (moderate-severe) IBD, or inactive IBD (remission) versus active IBD (mild, moderate, and severe). The ability of ELISA fMPOa, ELISA fMPOp, CM-S fMPOa, fCal, and urinary GSA to discriminate between different states of IBD severity were analyzed using ROC curves. The maximal Youden index was calculated to identify the optimal cutoff point for each biomarker for discriminating between different disease states.^25^ The ROC curves (AUROCs) were compared as paired samples as described by DeLong et al.^26^ These analyses were performed using IBM SPSS Statistics SPSS (version 29.0, IBM, Armonk, NY, USA). Logistic regressions were conducted on combinations of biomarkers and/or symptoms and the predicted probabilities from these models were then summarized as ROC curves. The combinations that resulted in a higher AUROC than the best individual biomarker were then statistically compared with the best solo biomarker. The maximum specificity/sensitivity of these combinations was identified from the maximum Youden index. A 2-tailed *P*-value < .05 is taken to indicate statistical significance.

All authors had access to the study data and have reviewed and approved the final submitted manuscript.

## Results

### Study Population

The final NIDA-IBD cohort consisted of 172 participants (ulcerative colitis *n* = 72, Crohn’s disease *n* = 100) who had completed the study questionnaires and provided biological samples. Demographics and details of this cohort have been described previously.^3^ Descriptive statistics pertinent to the biomarkers assessed in this study are summarized in Supplementary Table S2.

### Correlations Between ELISA-Measured fMPO Enzyme Activity With fMPO Protein Concentration and other IBD Biomarkers

**Figure 1.**
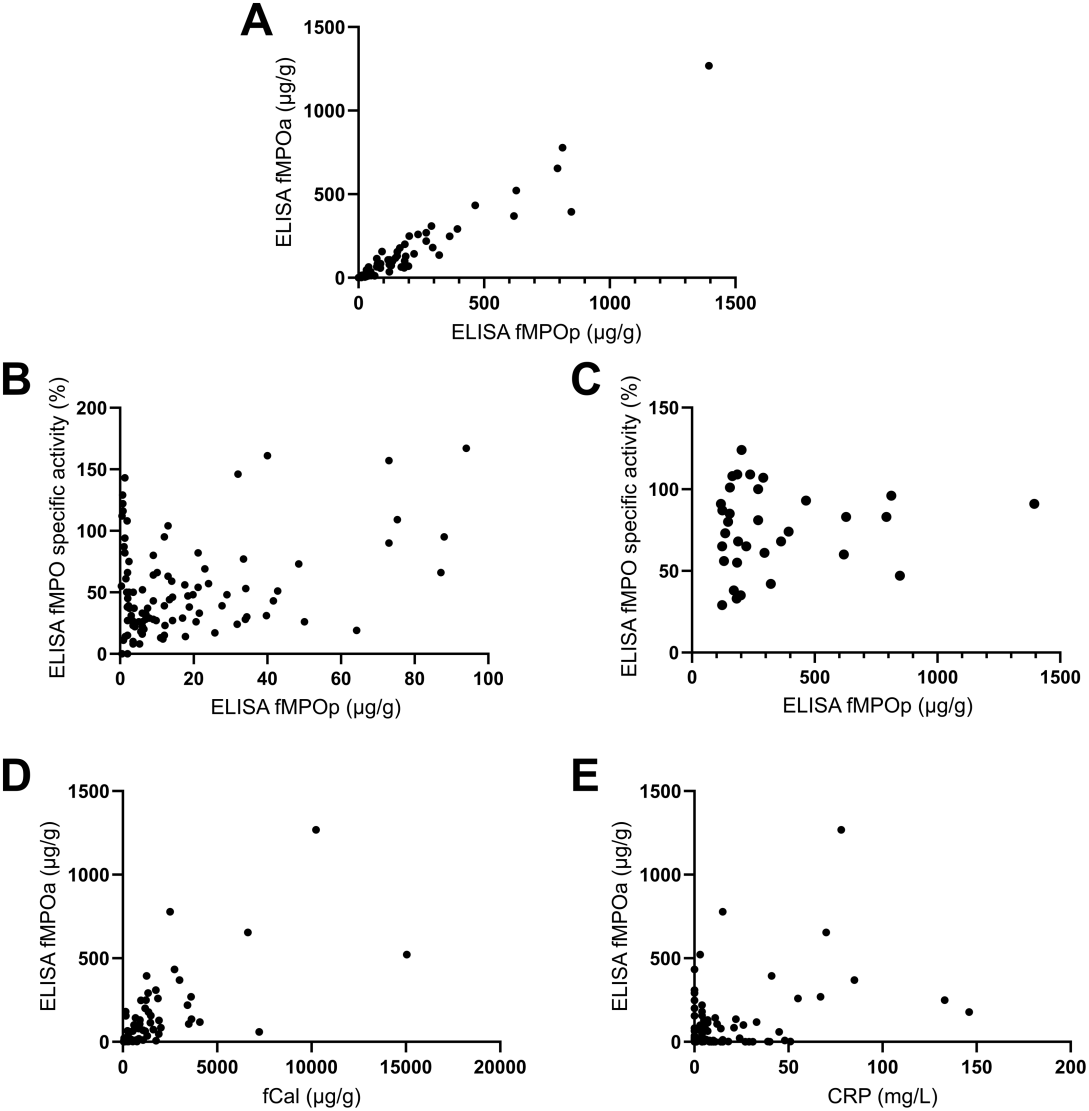
ELISA fMPOa correlates with ELISA fMPOp, fCal, and CRP. (A) ELISA fMPOa significantly correlates with ELISA fMPOp (*n* = 172) (B–C) ELISA fMPO specific activity (% fMPOa/fMPOp) in samples with (B) ELISA fMPOp < 100 μg/g (*n* = 101), and (C) ELISA fMPOp > 100 μg/g (*n* = 33). (C) Correlation of ELISA fMPOa and fCal (*n* = 172). (D) Correlation between ELISA fMPOa and CRP (*n* = 172). Statistical analysis was conducted on GraphPad Prism. Abbreviations: CRP, c-reactive protein; ELISA fMPOa, ELISA-measured fecal myeloperoxidase activity; ELISA fMPOp, ELISA-measured fecal myeloperoxidase protein; fCal, fecal calprotectin.

The performance of ELISA fMPOa was evaluated by comparing it to ELISA fMPOp measured within the same assay. We have previously reported data for MPO protein measurements.^3^ Now we report the recovery of spiked myeloperoxidase protein in comparison with its activity (Supplementary Figure S1). Protein measurements were in good agreement with the values obtained previously (previous fMPOp mean = 77 μg/g; standard deviation (SD) = 177 μg/g; remeasured fMPOp mean = 73 μg/g, SD = 177 μg/g: *P* > .05).^3^ ELISA fMPOa and ELISA fMPOp strongly correlated with each other (*r = *0.91, *P < .*0001) (Figure 1A). The specific activity of detectable fMPO showed that ELISA fMPOa was consistently lower than equivalent fMPOp. The average specific activity was 58 ± 36% (SD) (Figure 1B and C). In samples with less than 100 μg/g ELISA fMPOp, the average specific activity was 52 ± 37% (SD) (Figure 1B), while in samples with greater than 100 μg/g ELISA fMPOp, the specific activity was higher at an average of 78 ± 29% (SD) (Figure 1C). It should be noted that a higher degree of uncertainty applies to the specific activity at low concentrations of fMPO (eg, < 15 μg/g), due to the ratio involving smaller numbers with comparatively larger errors. Samples with lower fMPO content were more likely to have relatively less enzyme activity. This could be due in part to the degradation of myeloperoxidase in the feces, as ELISA fMPOa was negatively affected following one day of storage at 4 °C in solid feces (Supplementary Figure S2). However, when solubilized into CTAB buffer, ELISA fMPOa was stable for up to 3 days at 4 °C (Supplementary Figure S2).

ELISA fMPOa significantly correlated with fCal (*r = *0.73, *P < .*0001) (Figure 1D). This correlation was similar to previously reported correlations of ELISA fMPOp and fCal (r = 0.82, *P < .*001).^3^ There was a weaker, but significant relationship between ELISA fMPOa and CRP (*r = *0.31, *P < .*0001) (Figure 1E).

### Development of the CM-Sepharose Extraction Assay to Measure fMPO Enzyme Activity

**Figure 2.**
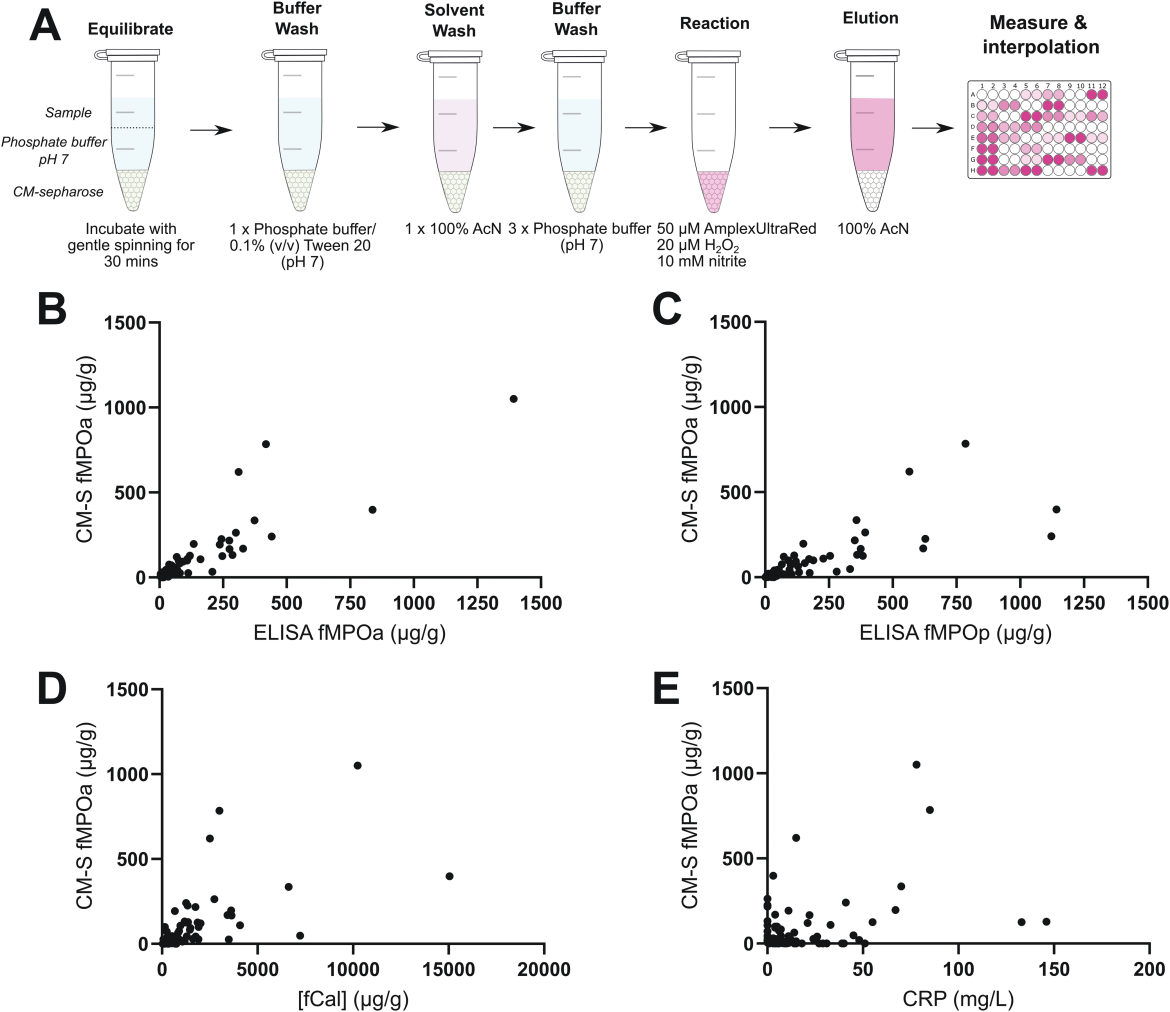
Overview of the CM-S fMPOa assay and correlations with biomarkers. (A) A schematic overview of the CM-S fMPOa assay method. (B–E) Correlations of CM-S fMPOa compared to (B) ELISA fMPOa, (C) ELISA fMPOp, (D) fCal, or (E) CRP. (B–E) Spearman’s correlations calculated in GraphPad Prism. For B–E, (*n* = 172). Abbreviations: CM-S fMPOa, CM-sepharose extraction assay measured fecal myeloperoxidase activity; CRP, c-reactive protein; ELISA fMPOa, ELISA-measured fecal myeloperoxidase activity; ELISA fMPOp, ELISA-measured fecal myeloperoxidase protein; fCal, fecal calprotectin.

We developed an alternative antibody-independent assay that could measure fMPO enzyme activity. Solubilized fMPO was captured using the cation exchange resin CM-Sepharose (Figure 2A, Supplementary Methods). Development of the CM-sepharose myeloperoxidase extraction assay was complicated by the co-purification of unidentified interfering compounds from feces. The effect of these interfering compounds was minimized by several adjustments, including conducting the assay at pH 7, the use of a Tween20-containing wash buffer, and a solvent wash step using 100% acetonitrile (Figure 2A, Supplementary Figure S4). When conducted at pH 7, oxidation of the AmplexUltraRed mixture by myeloperoxidase was also optimized, with minimal background fluorescence (Supplementary Figure S4A and B). Washing the fecal samples extracted by CM-sepharose with 100% acetonitrile or 96% ethanol removed interfering compounds from the subsequent enzyme reaction (Supplementary Figure S4D). Additionally, the acetonitrile wash step, which was subsequently followed by buffer washes, improved the signal of the subsequent colorimetric reaction, whereas ethanol did not (Supplementary Figure S4C).

To estimate myeloperoxidase recovery in the CM-sepharose extraction assay, purified human myeloperoxidase was spiked into 10 solubilized fecal samples with negligible ELISA fMPO levels, or into an equivalent buffer solution, and then processed simultaneously by the fMPO ELISA or the CM-S fMPOa assay. Recovery of spiked myeloperoxidase was 92% ± 24% (SD) by the CM-S fMPOa assay, 92% ± 20% (SD) by ELISA fMPOa, and 99% ± 18% (SD) by ELISA fMPOp (Supplementary Figure S4E).

### Specificity of the CM-Sepharose Extraction Assay

Selective peroxidase inhibitors were used to investigate the specificity of the CM-S fMPOa assay.^24^ Seven fecal samples that had CM-S fMPOa greater than ELISA fMPOa were used in the selective inhibitor experiment (average specific activity of CM-S fMPOa/ELISA fMPOa = 166% ± 27% (SD). The samples were processed in parallel in the ELISA and CM-S assay, and were pre-incubated with H_2_O_2_ alone, or H_2_O_2_ together with either dapsone, which inhibits eosinophil peroxidase and lactoperoxidase but not myeloperoxidase, or the myeloperoxidase-selective inhibitor AZM198, prior to subsequent activity measurement (Supplementary Figure S4F–G).^24^ Dapsone mildly inhibited ELISA fMPOa, whereas, in the CM-S fMPOa assay, dapsone increased the signal in fecal samples (Supplementary Figure S4F–G). In both assays, AZM198 inhibited the oxidation of AmplexUltraRed (Supplementary Figure S4F–G). Thus, the CM-S fMPOa assay appeared selective for fMPO activity.

### Comparison of the CM-Sepharose Extraction Assay to the fMPO ELISA and Other Biomarkers

A summary of the optimized CM-sepharose extraction assay to measure fMPO activity is shown in Figure 2A. The CM-S fMPOa assay was compared to the ELISA fMPO assay to evaluate its measurement of fMPO activity. Fecal samples were processed in parallel by the ELISA fMPO assay or by the CM-S fMPOa assay. CM-S fMPOa correlated with ELISA fMPOa (*r = *0.89, *P < .*0001) and ELISA fMPOp (*r = *0.79, *P < .*0001) (Figure 2B and C). This confirmed that the CM-S fMPOa assay performed similarly to ELISA-measured fMPOa or fMPOp. CM-S fMPOa significantly correlated with fCal (*r = *0.71, *P < .*0001) and CRP (*r = *0.33, *P < .*0001) (Figure 2D–F).

### Associations of ELISA fMPO Activity With Endoscopic Activity in IBD

**Figure 3.**
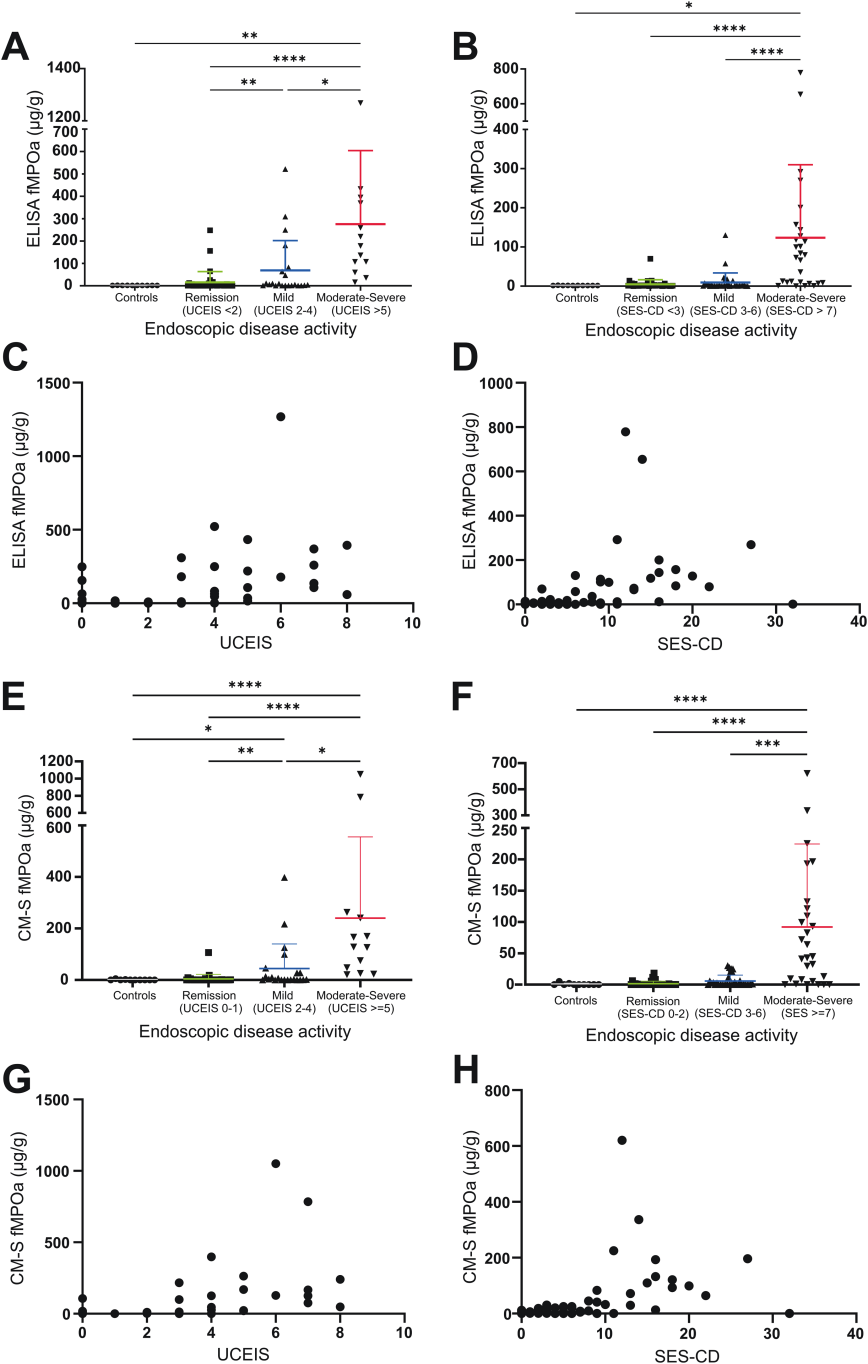
Associations of ELISA fMPOa and CM-S fMPOa with endoscopic disease activity scores of IBD. (A–B) Subgroup analysis of ELISA fMPOa measured in fecal samples from healthy controls (*n* = 10) and (A) ulcerative colitis (*n* = 72) or (B) Crohn’s disease (*n* = 100) patients with their disease assessed by either the UCEIS or the SES-CD. Correlations of ELISA fMPOa and disease scores; (C) UCEIS, (D) SES-CD. (E–H) Subgroup analysis of CM-S fMPOa measured in healthy controls (*n* = 10), (E) ulcerative colitis (*n* = 72) or (F) Crohn’s disease (*n* = 100) patients, who had their disease assessed by either the UCEIS or the SES-CD. (G–H) Correlations of CM-S fMPOa with either the (G) UCEIS or the (H) SES-CD. (A–B, E–F) Data was analyzed using GraphPad Prism to conduct Kruskal–Wallis test, with Dunne’s planned multiple comparisons between each group. Bars represent mean ± SD. *P < .05, **P < .01, ***P < .001, ****P < .0001 (C–D, G–H) Spearman’s correlations calculated in GraphPad Prism. CM-S fMPOa, CM-sepharose extraction assay measured fecal myeloperoxidase activity; ELISA fMPOa, ELISA-measured fecal myeloperoxidase activity; IBD, inflammatory bowel disease; SES-CD, simple endoscopic score for Crohn’s disease; UCEIS, ulcerative colitis endoscopic index of severity.

Subgroup analysis was conducted using IBD grouped by endoscopic disease activity. In ulcerative colitis, ELISA fMPOa was significantly elevated in moderate-severe ulcerative colitis compared to patients with mild disease (*P < .*05), those in remission (*P < .*0001) or healthy controls (*P < .*01) (Figure 3A). ELISA fMPOa was also significantly elevated in patients with mild ulcerative colitis compared to patients in remission (*P < .*001) (Figure 3A). Similarly, in Crohn’s disease, ELISA fMPOa was significantly elevated in patients with moderate-severe Crohn’s disease compared to patients who had mild disease (*P < .*0001), to those in remission (*P < .*0001), or to healthy controls (*P < .*05) (Figure 3B). When stratified into endoscopically inactive (UCEIS < 2, SES-CD < 3) or endoscopically active (UCEIS > 2, SES-CD > 3) disease, ELISA fMPOp and ELISA fMPOa were both significantly elevated in endoscopically active ulcerative colitis (*P* < .0001) and Crohn’s disease (*P* < .01) (Supplementary Figures S5A and B and S6A and B).

Correlations between ELISA fMPOa and measures of IBD severity were investigated to further assess the utility of ELISA fMPOa in IBD. ELISA fMPOa correlated with IBD severity measures similarly to fCal and ELISA fMPOp (Table 1). ELISA fMPOa significantly correlated with UCEIS score, SCCAI, and Mayo score in ulcerative colitis (Table 1, Figure 3C, Supplementary Figure S7). For Crohn’s disease, ELISA fMPOa significantly correlated with endoscopic disease activity as assessed by the SES-CD and CDAI scores but did not correlate with symptoms as assessed by the HBI (Table 1, Figure 3D, Supplementary Figure S7). When these correlations were conducted only in individuals with endoscopically active disease (UCEIS > 1, SES-CD > 2), ELISA fMPOa correlated with all clinical scores in ulcerative colitis and the SES-CD score, but did not significantly correlate with CDAI or HBI scores (Supplementary Table S3). In a previous analysis of this cohort, both HBI and CDAI did not correlate with SES-CD score.^3^

**Table 1.** Spearman’s correlations of fecal biomarkers with inflammatory bowel disease clinical scores.

Biomarkers	IBD clinical scores					
	UCEIS (*n* = 72)	Mayo score (*n* = 72)	SCCAI (*n* = 72)	SES-CD (*n* = 100)	CDAI (*n* = 100)	HBI (*n* = 100)
**ELISA fMPOa**	0.65^*^	0.65^*^	0.56^*^	0.51^*^	0.21^*^	0.20
**ELISA fMPOp**	0.62^*^	0.64^*^	0.51^*^	0.51^*^	0.15	0.20^*^
**CM-S fMPOa**	0.65^*^	0.71^*^	0.61^*^	0.58^*^	0.14	0.17
**fCal**	0.67^*^	0.71^*^	0.62^*^	0.51^*^	0.10	0.03
**CRP**	0.50^*^	0.53^*^	0.51^*^	0.36^*^	0.20^*^	0.19

^*^= statistically significant (*P* < .05). Abbreviations: CDAI, Crohn’s disease activity index; CM-S fMPOa, CM-sepharose extraction assay measured fecal myeloperoxidase activity; CRP, C-reactive protein; ELISA fMPOa, ELISA-measured fecal myeloperoxidase activity; ELISA fMPOp, ELISA-measured fecal myeloperoxidase protein; fCal, fecal calprotectin; HBI, Harvey-Bradshaw index; SCCAI, simple clinical colitis activity index; SES-CD, simple endoscopic score for Crohn’s disease; UCEIS, ulcerative colitis endoscopic index of severity. Data for fecal calprotectin and CRP were published previously.^3^.

### Associations of CM-S fMPOa With Measures of IBD Severity

CM-S fMPOa was then examined for its association with endoscopic disease severity in IBD by subgroup analysis and correlations with disease scores. CM-S fMPOa was significantly elevated with more severe ulcerative colitis and Crohn’s disease (Figure 3E and F). CM-S fMPOa was significantly elevated in moderate-severe ulcerative colitis compared to individuals with mild ulcerative colitis (*P < .*05), individuals in remission (*P < .*0001), or healthy controls (*P < .*0001) (Figure 3E). Patients with mild ulcerative colitis also had elevated CM-S fMPOa in comparison to individuals in remission (*P < .*01) or healthy controls (*P < .*05) (Figure 3E). In Crohn’s disease, CM-S fMPOa was significantly elevated in individuals with moderate-severe endoscopic severity in comparison to individuals with mild activity (*P < .*001), to those in remission (*P < .*0001), and to healthy controls (*P < .*0001) (Figure 3F). In patients stratified to either endoscopically inactive or active disease, CM-S fMPOa was significantly elevated in patients with active ulcerative colitis (*P* < .0001), and in patients with active Crohn’s disease (*P* < .0001) (Supplementary Figure S5C and S6C).

CM-S fMPOa significantly correlated with UCEIS score (*r = *0.65, *P < .*0001) and SES-CD score (*r = *0.56, *P < .*0001) (Figure 3G and H, Table 1). CM-S fMPOa significantly correlated with ulcerative colitis symptoms as assessed by the SCCAI score (*r = *0.61, *P < .*0001) and Mayo clinical scores (*r = *0.71, *P < .*0001) (Table 1, Supplementary Figure S8). In comparison, symptoms assessed by the HBI and the CDAI did not correlate with CM-S fMPOa (HBI: *r = *0.17, *P = .*09; CDAI: *r = *0.14, *P = .*18) (Table 1, Supplementary Figure S8) in Crohn’s disease patients. Correlations for CM-S fMPOa and these clinical scores in individuals with endoscopically active disease behaved similarly to correlations performed on the whole cohort (Supplementary Table S3). We have previously assessed the performance of fCal and CRP as biomarkers in this cohort.^3^

### Associations of Urinary GSA With Endoscopic Activity and Biomarkers in IBD

**Figure 4.**
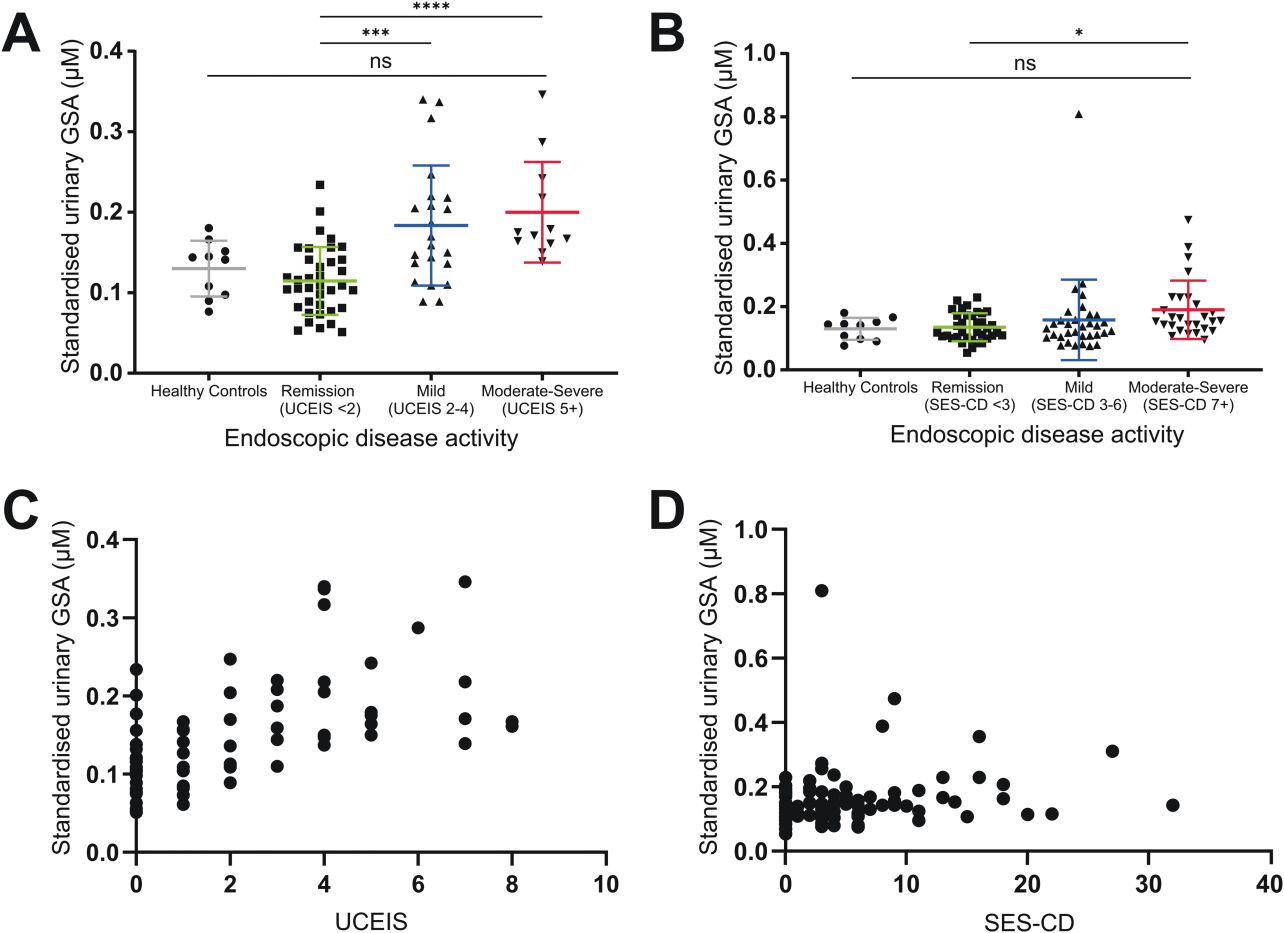
Urinary GSA is significantly elevated with more severe ulcerative colitis and Crohn’s disease. Urinary GSA standardized to specific gravity was measured in healthy controls, and in individuals with IBD with their disease activity assessed by colonoscopy. (A–B) Subgroup analysis of urinary GSA in IBD, grouped by disease activity assessed by either the UCEIS or the SES-CD. Urinary GSA in (A) ulcerative colitis (*n* = 71) and (B) Crohn’s disease (*n* = 99). Data analyzed by Kruskal–Wallis non-parametric test, with Dunn’s multiple comparison’s test. *P < .05, ***P < .001, ****P < .0001. Bars represent mean ± SD. Spearman’s correlations calculated between the relationship of urinary GSA and (C) the UCEIS in individuals with ulcerative colitis (*n* = 71) and (D) the SES-CD in individuals with Crohn’s disease (*n* = 99). Statistical analysis completed in GraphPad Prism. Abbreviations: GSA, glutathione sulphonamide; IBD, inflammatory bowel disease; SES-CD, simple endoscopic score for Crohn’s disease; UCEIS, ulcerative colitis endoscopic index of severity.

Urinary GSA was investigated as an indirect measure of myeloperoxidase activity in IBD to assess whether myeloperoxidase produced HOCl during inflammation. Standardized urinary GSA was quantified in healthy controls and individuals from the NIDA-IBD cohort (Figure 4A and B). GSA was detected in the urine of healthy controls and individuals with IBD (Figure 4A and B, Supplementary Table S2). Subgroup analysis showed that urinary GSA performed slightly better in ulcerative colitis compared to Crohn’s disease. There was a significant difference between urinary GSA in individuals with moderate-severe (*P < .*0001) and mild ulcerative colitis (*P < .*001) when compared to individuals in remission (Figure 4 A). Urinary GSA was significantly elevated in individuals with moderate-severe Crohn’s disease compared to patients in endoscopic remission (*P < .*05) (Figure 4B). There was no statistically significant difference in the levels of urinary GSA in healthy controls compared to individuals with moderate-severe or mild IBD endoscopic activity (Figure 4A and B). When endoscopic disease activity was grouped as either inactive or active disease, urinary GSA was significantly elevated in patients with active ulcerative colitis (*P* < .0001) (Supplementary Figure S5D). There was no statistically significant difference in urinary GSA levels when comparing active and inactive Crohn’s disease (*P* = .108) (Supplementary Figure S6D).

Urinary GSA correlated moderately with UCEIS score (*r = *0.61, *P < .*0001) (Figure 4C, Table 1). In comparison, the correlation of urinary GSA and Crohn’s disease endoscopic activity, while statistically significant, was weaker (*r = *0.28, *P = .*005) (Figure 4D, Table 1). Urinary GSA correlated with clinical and symptomatic scores in ulcerative colitis, but not in Crohn’s disease (Table 1, Supplementary Figures S9 and S10). In individuals with endoscopically active disease, urinary GSA did not correlate with the UCEIS score (*r = *0.34, *P = .*05), but did correlate with the SES-CD score (*r = *0.27, *P < .*05) (Supplementary Table S3).

The relationship between urinary GSA and other biomarkers of IBD were separately evaluated in ulcerative colitis and Crohn’s disease (Supplementary Figures S9 and S10). In patients with ulcerative colitis, urinary GSA correlated with ELISA fMPOp (*r = *0.45, *P < .*0001), ELISA fMPOa (*r = *0.45, *P < .*0001), fCal (*r = *0.61, *P < .*0001) and CRP (*r = *0.29, *P = .*01) (Supplementary Figure S9). While the correlations were not as strong in Crohn’s disease, urinary GSA significantly correlated with other biomarkers, including ELISA fMPOp (*r = *0.25, *P = .*01), ELISA fMPOa (*r = *0.27, *P < .*01), fCal (*r = *0.33, *P < .*01) and CRP (*r = *0.29, *P < .*01) (Supplementary Figure S10). These results suggest that in ulcerative colitis myeloperoxidase is producing HOCl at the site of inflammation, while in Crohn’s disease, the link with myeloperoxidase activity and inflammation is less clear.

### ROC Analyses of Examined Biomarkers in Discriminating Endoscopic Activity

**Figure 5.**
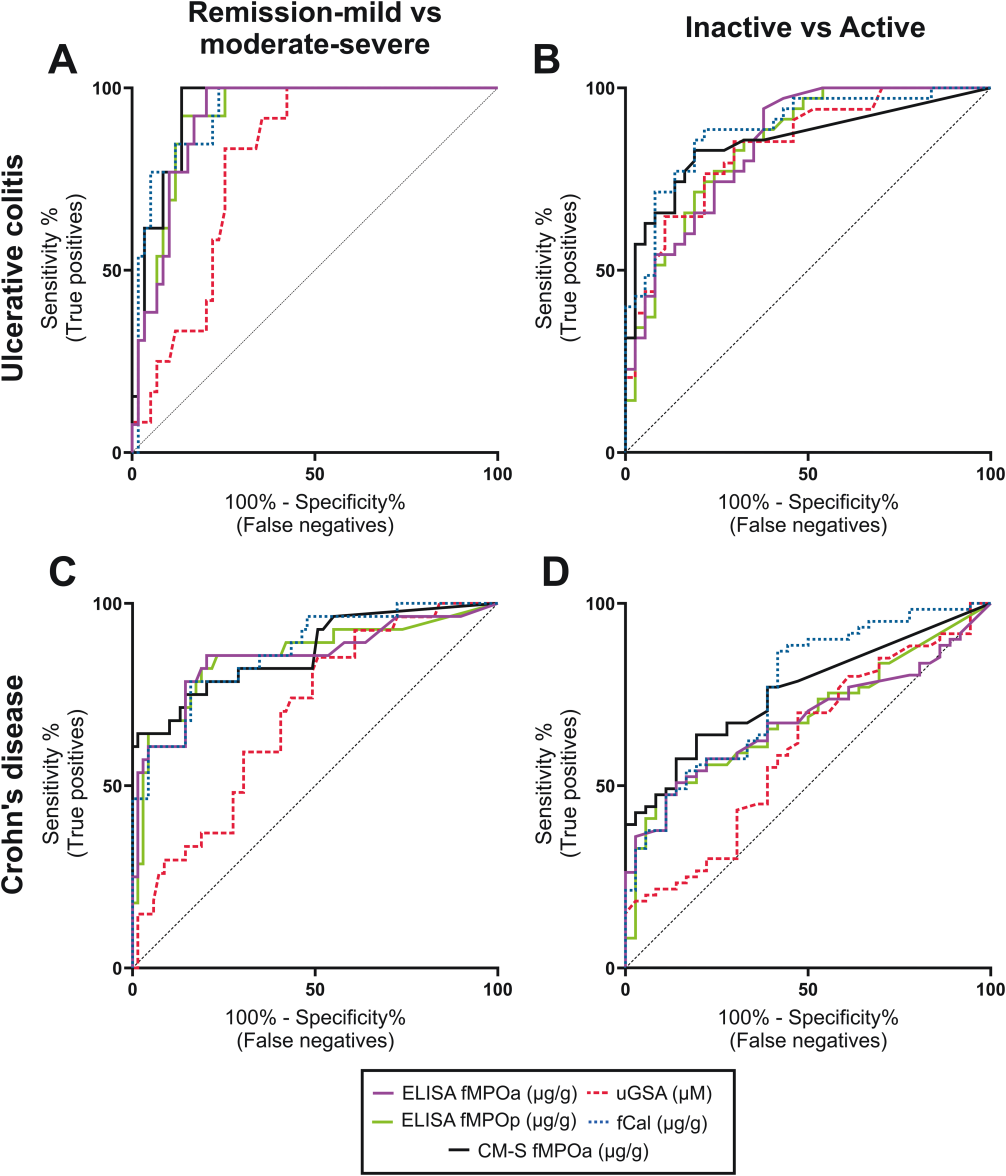
ROC curve evaluation of faecal myeloperoxidase and urinary GSA as biomarkers of IBD severity. IBD endoscopic activity was assessed by the UCEIS for ulcerative colitis or the SES-CD for Crohn’s disease. ELISA fMPOa, ELISA fMPOp, urinary GSA (uGSA), fCal, and CM-S fMPOa were evaluated for their ability to distinguish between (A, C) remission-mild or moderate-severe (A) ulcerative colitis or (C) Crohn’s disease. (B, D) These biomarkers were also evaluated for their ability to discriminate between inactive and active (B) ulcerative colitis or (D) Crohn’s disease. Data for fCal were published previously.^3^ Statistical analyses conducted on SPSS, graphs created in GraphPad Prism. Abbreviations: CM-S fMPOa, CM-sepharose extraction assay measured fecal myeloperoxidase activity; ELISA fMPOa, ELISA-measured fecal myeloperoxidase activity; ELISA fMPOp, ELISA-measured fecal myeloperoxidase protein; GSA, glutathione sulphonamide; fCal, fecal calprotectin; GSA, glutathione sulphonamide; IBD, inflammatory bowel disease; ROC, receiver operator characteristic; SES-CD, simple endoscopic score for Crohn’s disease; UCEIS, ulcerative colitis endoscopic index of severity.

The biomarkers examined in this study were assessed by ROC curves to determine their ability to discriminate IBD endoscopic severity (Figure 5, Table 2). These showed that the AUROCs of the fecal biomarkers (fCal, ELISA fMPOa, ELISA fMPOp, CM-S fMPOa) were not significantly different from each other in any comparison. In each condition, fCal had the highest AUROC (Table 2).

**Table 2.** ROC curve analysis and optimum cutoff values for each biomarker in discriminating inflammatory bowel disease disease activity.

Disease	Disease grouping	Biomarker	AUROC	cutoff (μg/g, mg/L or score)	Sensitivity (%)	Specificity (%)
**Crohn’s disease**	**Remission-mild vs moderate-severe**	fCal	0.86	252	78	84
		ELISA fMPOa	0.85	6.3	85	80
		ELISA fMPOp	0.85	13.6	82	81
		CM-S fMPOa	0.86	28.3	63	99
		uGSA	0.69	0.1215	85	49
		CRP	0.69	3.5	67	67
		HBI	0.53	14.5	23	96
	**Inactive vs active**	fCal	0.76	58.2	87	60
		ELISA fMPOa	0.68	6.9	49	86
		ELISA fMPOp	0.68	19.3	46	91
		CM-S fMPOa	0.76	0.8	64	79
		uGSA	0.6	0.1195	69	51
		CRP	0.66	3.5	53	75
		HBI	0.51	9.5	36	86
**Ulcerative colitis**	**Remission-mild vs moderate-severe**	fCal	0.94	270	100	76
		ELISA fMPOa	0.92	14.2	100	80
		ELISA fMPOp	0.92	44.3	92	86
		CM-S fMPOa	0.94	19.6	100	86
		uGSA	0.8	0.16	83	75
		CRP	0.8	10.5	58	93
		SCCAI	0.91	6.5	92	85
	**Inactive vs active**	fCal	0.88	137.5	88	78
		ELISA fMPOa	0.84	0.95	94	62
		ELISA fMPOp	0.84	4.4	85	68
		CM-S fMPOa	0.85	0.65	83	81
		uGSA	0.84	0.134	85	70
		CRP	0.72	3.5	62	78
		SCCAI	0.86	3.5	88	78

Abbreviations: CM-S fMPOa, CM-sepharose extraction assay measured fecal myeloperoxidase activity; CRP, c-reactive protein; ELISA fMPOa, ELISA-measured fecal myeloperoxidase activity; ELISA fMPOp, ELISA-measured fecal myeloperoxidase protein; fCal, fecal calprotectin; HBI, Harvey-Bradshaw index; ROC, receiver operator characteristic; SCCAI, simple clinical colitis activity index; uGSA, urinary glutathione sulphonamide. Data for fecal calprotectin, CRP, the HBI, and the SCCAI were published previously.^3^

ELISA fMPOa, ELISA fMPOp, and CM-S fMPOa showed good discriminatory ability in differentiating between remission-mild and moderate-severe, or inactive and active ulcerative colitis (Figure 5A and C) and Crohn’s disease (Figure 5B and D, Table 2). Urinary GSA did not perform as well as the fecal biomarkers in discriminating between less severe and more severe ulcerative colitis (AUROC = 0.69, *P < .*05 for urinary GSA compared to each fecal biomarker) (Figure 5A, Table 2). Urinary GSA performed similarly to the fecal biomarkers in discriminating between inactive and active disease in ROC-curve analysis (no significant difference between urinary GSA AUROC and other biomarkers) (Figure 5B and D).

When discriminating between remission-mild and moderate-severe Crohn’s disease, or inactive and active Crohn’s disease, the fecal markers performed similarly well (Figure 5C and D, Table 2). In remission-mild compared to moderate-severe Crohn’s disease, urinary GSA did not perform as well as the fecal biomarkers (*P < .*05). In inactive compared to active Crohn’s disease, the performance of urinary GSA was significantly poorer compared with fCal (*P = .*02) and CM-S fMPOa (*P = .*02) but was not significantly different to ELISA-measured fMPO activity or protein.

The optimal discriminatory cutoff values for ELISA fMPOa or CM-S fMPOa in the tested disease states (inactive vs active or remission-mild vs moderate-severe) were all low (Table 2). This suggested that even minor fMPO activity was associated with active and more severe IBD, especially when compared with the much higher optimum cutoff values calculated for fCal (Table 2). The practical use of urinary GSA as a biomarker in ulcerative colitis would likely be affected by the substantial signal of urinary GSA in healthy controls and those in endoscopic remission (Figure 4A and B).

### Combination of Myeloperoxidase Activity Measures and Other Measures of IBD Severity

Combinations of biomarkers and clinical scores only gave a very subtle improvement (ie, higher AUROC scores) compared to the AUROC of fCal alone (Supplementary Table S4). None of these combinations were significantly better than fCal alone (Supplementary Table S4).

### Performance of Myeloperoxidase Activity Biomarkers in Isolated Ileal Crohn’s Disease

The ability of these biomarkers to reflect isolated ileal Crohn’s disease activity was assessed. In the NIDA-IBD cohort, 17 patients with Crohn’s disease had isolated ileal disease (L1 Montreal classification), 4 with inactive disease, and 14 with active disease determined by ileocolonoscopy or MRI (*n* = 1). Supplementary Table S5 and Supplementary Figure S11 summarize the median, interquartile range (IQR), and the Mann–Whitney *U* test comparison of the investigated biomarkers between inactive and active ileal disease patients. There were significantly different levels of CRP and CM-S fMPOa when comparing inactive and active ileal Crohn’s disease (Supplementary Table S5, Supplementary Figure S11). All other biomarkers were not statistically different between active and inactive isolated ileal Crohn’s disease.

## Discussion

We have extended our work on evaluating myeloperoxidase in feces as a biomarker of endoscopic disease severity in IBD by developing methods to measure its enzyme activity.^3^ We found that the enzyme activity of myeloperoxidase is linked to endoscopic gut inflammation in IBD. Both ELISA fMPOa and CM-S fMPOa were as effective as ELISA fMPOp and fCal at discriminating endoscopic disease activity in IBD. To understand whether myeloperoxidase had produced HOCl during inflammation in patients with IBD, we measured GSA in urine. Urinary GSA levels were more closely associated with endoscopic disease activity in ulcerative colitis than in Crohn’s disease. The association of myeloperoxidase’s enzyme activity with IBD activity suggests that it could be a useful biomarker, and its oxidants may have a pathological role in IBD.

Biomarkers are increasingly recognized as valuable tools for the management of IBD. This is reflected by the STRIDE-II guidelines, which state that normalization of biomarkers such as fCal is an appropriate intermediary target to monitor response to IBD treatment.^27^ A combination of CRP, fCal, or ELISA fMPOp and clinical symptoms have been shown to prognosticate long-term disease outcomes over 24 months.^28^ However, the practical use of implementing fCal into clinical care can be fraught with long turn-around times waiting for results of fCal ELISAs and the high cost of point-of-care devices.^29,30^ Both here and in our previous work we found that fCal and fMPO performed similarly in identifying endoscopically active IBD.^3^ By measuring fMPO enzyme activity and protein concentration in the same ELISA, we showed that fMPO retained most of its enzyme activity. Consequently, measurement of fMPO enzyme activity could be developed into a fast low-cost test for endoscopically active IBD, as we have shown by our proof-of-concept CM-S fMPOa assay.

Measuring the enzyme activity of fMPO instead of its protein concentration would simplify the components required to test for this enzyme. The CM-sepharose assay does not require antibodies to purify myeloperoxidase from fecal samples and exploits the intrinsic enzyme activity of myeloperoxidase with the potential to deliver a sensitive point-of-care test for endoscopically determined inflammation in IBD. Myeloperoxidase and non-myeloperoxidase-selective inhibitors showed that the signal from the CM-S fMPOa assay was due to the presence of myeloperoxidase in the stool. Pretreatment of samples with the myeloperoxidase-specific inhibitor AZM198 blocked all signals in the CM-S fMPOa assay, while the non-myeloperoxidase peroxidase inhibitor dapsone did not inhibit activity. The unexpected rise in the fluorescent signal of dapsone-treated samples could indicate that in these assay conditions, dapsone acted as a radical shuttle and improved the oxidation of AmplexUltraRed by myeloperoxidase.^31^ Possible sources of non-myeloperoxidase peroxidase contamination in this assay could be further investigated by assessing the performance of this assay in individuals with eosinophilic enteritis. These individuals would likely have high levels of eosinophil peroxidase.

Removing interfering compounds from the CM-S fMPOa assay was key to ensuring adequate measurement of fMPO activity, as shown in Supplementary Figure S4D. However, given its toxicity, acetonitrile is not an ideal solvent for the implementation of a clinical test. Development of this assay into a clinical test would need to explore alternative, less-toxic solvents for their ability to remove interfering compounds from this assay. Other resins might be less susceptible to binding interfering compounds, which appear to adhere through non-polar interactions with the cation exchange resin.

An assay that measures solubilized fMPO activity immediately would likely provide the most accurate reading, as prolonged time in solid feces negatively affected ELISA fMPOa measurement compared to ELISA fMPOp (Supplementary Figure S2). However, storage in solubilization buffer at 4 °C preserved both ELISA fMPOa and ELISA fMPOp (Supplementary Figure S2). Therefore, solubilizing and measuring fecal samples as quickly as possible would give the best results of fMPO activity.

The practical advantages of measuring fMPO activity over fCal will need to be investigated by direct comparison with tests of fMPO activity that are ready to be used in the clinic. These advantages would likely occur by designing point-of-care tests that require fewer, cheaper, or more shelf-stable components than commercially available fCal tests, with a lower optimum cutoff value. The assays presented here are exploratory, proof-of-concept (CM-S fMPOa), or laboratory-based assays (ELISA fMPOa), and so do not currently have any advantages over fCal. However, our ROC-curve analysis shows that measuring fMPO activity with our current assays is as good as measuring fCal for detecting endoscopic disease activity.

Future research should directly correlate mucosal myeloperoxidase with fMPO protein concentration and enzyme activity. While this study showed that fMPO activity and protein concentration are linked to disease severity, the NIDA-IBD cohort did not provide mucosal samples for examination. Previous studies have shown that mucosal myeloperoxidase protein and peroxidase activity are elevated in individuals with IBD compared to controls.^32–34^

Patients with IBD have indicated that urinary biomarkers are preferred to fecal markers.^35^ A recent systematic review highlighted urinary biomarkers tested in IBD, though the focus for many of these studies was discrimination of individuals with IBD from healthy individuals.^36^ Here, we investigated elevated urinary GSA as an indirect measure of myeloperoxidase activity in IBD. As GSA is generated by the reaction of 3 molecules of HOCl with glutathione, elevated urinary GSA is suggestive of HOCl production occurring during inflammation.^13,15^ Urinary GSA levels correlated with markers of ulcerative colitis severity, including endoscopic score and neutrophil-derived fecal biomarkers. The association of urinary GSA and disease severity in Crohn’s disease was significant, but not as robust as in ulcerative colitis. The use of urinary GSA as a biomarker was unfortunately complicated by the high background of urinary GSA in healthy individuals and people in remission. However, elevated urinary GSA in moderate to severe IBD patients suggested that myeloperoxidase was producing HOCl during inflammation. Hence, other biomarkers should be explored to understand the possible pathological role of myeloperoxidase in IBD. One such marker could be 3-chlorotyrosine. It is specific to HOCl production and was found to be elevated in the mucosa and blood of individuals with active IBD.^37,38^

Measures of myeloperoxidase activity were more closely associated with disease activity in ulcerative colitis patients, with less robust correlations in Crohn’s disease. This is likely due to distinct pathophysiological differences between ulcerative colitis and Crohn’s disease. The role of neutrophils in ulcerative colitis and Crohn’s disease is complex. Ulcerative colitis has stereotypically been defined as a neutrophil-dominated disease compared to Crohn’s disease.^39^ In ulcerative colitis, active disease is associated with the presence of neutrophils in histological lesions and neutrophil-derived biomarkers.^40,41^ In comparison, one hypothesis of the cause of Crohn’s disease is inadequate bacterial clearance by neutrophils, which then leads to an excessive adaptive immune response. Neutrophil dysfunction, or inadequate recruitment of neutrophils has been observed in Crohn’s disease patients when compared to healthy controls or patients with ulcerative colitis.^39,42,43^

We found that many people with active Crohn’s disease lesions still had abnormally high levels of fMPO and fecal calprotectin, suggesting that neutrophils are a major feature of inflammatory lesions in most Crohn’s disease patients. Additionally, other studies have linked the presence of neutrophils to active Crohn’s disease, including complications like non-response to anti-TNF-α therapy and fistulas.^32,44–47^ It may be that there are subsets of Crohn’s disease patients whose disease is driven by neutrophil dysfunction. Future studies should investigate whether neutrophil-derived and non-neutrophil biomarkers could complement each other when assessing disease activity in Crohn’s disease.

Some limitations to these findings may exist due to low numbers in the NIDA-IBD cohort of individuals with moderate and severe IBD (Crohn’s disease 28%, ulcerative colitis 18%). Also, there were only 18 individuals recruited who had isolated ileal Crohn’s disease. The accuracy of the biomarkers we measured in ileal Crohn’s disease should be further investigated in cohorts with a larger proportion of this subset of patients. Another limitation of our study was that it lacked a central reading of endoscopic assessment. Also, recruited participants reflected a population of individuals with IBD, encompassing both patients with newer and more established diseases, as well as patients on various IBD therapies.^3^ However, a recent publication has shown that fMPO protein concentration is an excellent discriminatory marker between healthy or symptomatic controls and individuals with treatment-naïve IBD.^48^ Another study has shown that fMPO protein concentration could discriminate individuals with established IBD from those with IBS.^49^ Measures of fMPO activity should be validated in additional cohorts in order to better understand and generalize the results published here. As the NIDA-IBD cohort was recruited from a population predominantly of European descent in a developed nation, validation of these findings in other ethnicities and in emerging nations will be key to assessing the utility of these biomarkers.

The findings presented here provide a clear rationale to better understand the role of myeloperoxidase’s enzyme activity in the pathology of IBD. Future research should be aimed at determining whether myeloperoxidase is simply an innocent bystander or whether it actively contributes to neutrophil-mediated tissue damage during bouts of inflammation. It is also apparent from this work that an assay that simply extracts myeloperoxidase from feces and measures its enzyme activity could provide the basis of a cheap but accurate test for endoscopically active IBD.

## Supplementary Data

Supplementary data is available at *Inflammatory Bowel Diseases* online.

izaf109_suppl_Supplementary_Material

## Data Availability

All de-identified data presented in this study are available upon request by contacting the corresponding authors of this study.
