# Supplementary material for: Myeloperoxidase Enzyme Activity in Feces Reflects Endoscopic Severity in Inflammatory Bowel Disease
Source: Inflamm Bowel Dis. 2025 May 24;31(8):2254–68. doi: 10.1093/ibd/izaf109 (PMC12342798; doi:10.1093/ibd/izaf109)
Supplement: izaf109_suppl_Supplementary_Material [file izaf109_suppl_supplementary_material.docx]

# Supplementary

## Supplementary Materials

Human myeloperoxidase purified from blood was supplied by Planta Natural Products (Vienna, Austria) and was used for spiking experiments and to construct standard curves. The concentration per haem of 1 mg/ml stock was approximately 14 μM, as determined by measuring the absorbance of the enzyme at 430 nm (ϵ_430nm_ = 89,000 M^−1^cm^−1^).^1^ Bovine lactoperoxidase from milk, bovine catalase from liver, dapsone, Tween20, ExtraAvidin Alkaline Phosphatase and p-nitrophenylphosphate (PNP) were purchased from Sigma (St Louis, MO, USA). Bovine serum albumin (BSA) was sourced from Gibco via ThermoFisher (NZ). Hydrogen peroxide (H_2_O_2_) was sourced from LabServ supplied by ThermoFisher (Victoria, Australia) (ε_240nm_ = 43.6 M^−1^ cm^−1^). AmplexUltraRed and ethanol (96%, analytical reagent grade) was sourced from ThermoFisher (Victoria, Australia). AZM198, a specific myeloperoxidase inhibitor, was synthesized and provided as a kind gift from AstraZeneca (Mölndal, Sweden). RIDA®TUBE vials were from R-Biopharm AG (Darmstadt, Germany). CM-Sepharose Fast Flow cation exchange resin (CM-Sepharose) was supplied by GE Healthcare (Uppsala, Sweden). High-binding 96-well plates were from Costar (Washington, DC, USA). Acetonitrile (HPLC grade, 100%) was supplied by Fisher Chemical (Fairlawn, NJ, USA). EDTA-vacutainers were from Becton Dickinson (Franklin Lakes, NJ, USA). All other materials were sourced from Sigma/Merck (Darmstadt, Germany) or BDH Laboratory Supplies (Poole, England). The antibodies used in the myeloperoxidase ELISA are detailed in the ELISA Supplementary methods.

## Supplementary Methods

*NIDA-IBD biological sample (blood, urine and faeces) collection:*

Before their ileocolonoscopy, recruited IBD patients were given kits for collecting faeces and urine. These kits were provided in insulated bags included sample collection pottles for urine and faecal samples, as well as gloves and zip-loc bags/a disposable container to store the samples, and a freezer pack. Participants were advised to take a faeces & urine sample prior to beginning bowel preparation for their ileocolonoscopy. Participants could then store their samples in a provided insulated bag with the freezer pack before delivering them to a community laboratory collection centre within Canterbury (either Canterbury Health Laboratories, or Southern Community Laboratories). The insulated bag with the freezer pack was anticipated to keep samples cool at approximately 4°C in between transit to the collection centre. Laboratory staff were asked to refrigerate samples at 4°C until received by study investigators. Study investigators then collected these packs for processing at room temperature before eventual storage at -80°C. If study investigators needed to temporarily store the samples before processing, the samples were kept refrigerated at 4°C.

At the time of ileocolonoscopy, blood was drawn through the intravenous cannula inserted as a routine part of the patient’s index investigation, using a vacutainer. The vacutainer blood tubes collected were one 9 ml lithium heparin tube, and two 3 ml ethylenediaminetetraacetic acid (EDTA) tubes. The lithium heparin tube, and one of the EDTA tubes were delivered to Canterbury Health Laboratories for automated processing of full blood count, serum creatinine, albumin and C-reactive protein. The other EDTA tube was retained by the study investigators for biobanking plasma for biomarker investigation unrelated to this manuscript.

*NIDA-IBD biological sample (urine and faeces) processing:*

Faecal, urine, and plasma samples were processed and stored at -80°C within 48 hours of arriving at the research laboratory. All sample handling was inside a biological containment hood using sterile techniques. Samples were stored in 1.7 ml micro-centrifuge tubes and labelled with the participant’s identifier number.

For each whole faeces received, several samples were taken from different regions of the faeces for each stored aliquot. This was so that not only interior or exterior regions were selected for, as biomarkers may be distributed unequally through the faeces. These whole faecal samples were manipulated using wooden applicators, or sterile Pasteur pipettes if liquid and unable to be manipulated with wooden applicators.

Additionally, upon being received, whole faeces samples were solubilised using the Calpro Easy Extract device (Calpro AS, Norway) for the faecal calprotectin ELISA.

Urine samples were mixed, and then aliquoted using a sterile Pasteur pipette.

Samples were processed and then stored at -80°C as soon as possible. However, due to transit time between sample drop-off centres and the study investigators' laboratory, there could be up to four days delay between sampling and processing/storage. The samples were anticipated to be at around 4°C during this transit time, however it was not possible to know whether the samples were stored at higher temperatures than this. Unfortunately, the time that samples were produced by participants and then processed for storage was not consistently recorded during this study. Standard operating procedures for future cohort recruitment studies have been updated to systematically record sampling collection time and processing time.

*fMPO ELISA:*

Whole faecal samples were stored at -80°C until analysis. Faecal samples were thawed at room temperature and homogenized with a wooden stick. 10 mg of faeces were solubilized into 1 ml of CTAB faecal solubilization buffer (0.2% (w/v) CTAB, 0.0025% (v/v) Tween20, 1% BSA in phosphate buffered saline (PBS)) using a RIDA® Tubes (R-Biopharm AG, Germany). Captured faeces were soaked in the solubilization buffer for 20 minutes, and samples were vortexed for three minutes until turbid. A standard curve of human purified myeloperoxidase (Planta Natural Products, Austria) varying between 0.7 ng/ml to 50 ng/ml was used on each plate. Faecal samples were diluted a minimum of 1/10 in assay buffer (0.0025% (v/v) Tween20, 1% BSA in PBS) before being added to the ELISA plate; samples with high fMPO were diluted further until they fell within the linear segment of the standard curve. All washes were conducted three times using PBS.

The primary capture antibody used on the plate was BioRad (0400-0002) 4A4 anti-myeloperoxidase antibody diluted to 1 μg/ml (1/1000). A high-binding 96 well plate (Costar, USA), was incubated with 50 μl/well of primary capture antibody diluted in PBS overnight at room temperature. The plate was then washed and blocked with 75 μl/well assay buffer for up to two hours at room temperature. After blocking, the plate was washed, and standards and samples were added to the plate at 50 μl/well in technical duplicate and incubated for one hour at 37°C. After washing, myeloperoxidase enzyme activity was measured. The reaction mixture containing 50 mM NaBr/20 μM H_2_O_2_/50 μM AmplexUltraRed in 50 mM phosphate buffer (pH 7.4) was added to the plate at 50 μl/well. The development of a pink-fluorescent product with excitation and emission wavelengths of 544 nm and 590 nm respectively were measured every five minutes for up to 20 minutes using a Synergy Neo2 (BioTek, USA) plate reader. The time point with the most linear standard curve was chosen for analysis.

The activity reaction mixture was washed from wells, and myeloperoxidase protein concentration was then measured. A rabbit polyclonal anti-myeloperoxidase serum, (RAMPO, produced in house)^2^, diluted 1/800 in assay buffer was added to the plate at 50 μl/well and left to incubate overnight at 4°C. The following morning the plate was washed and incubated at 37°C for one hour with 50 μl/well of biotinylated polyclonal goat anti-rabbit antibody (GAR, Dako), diluted 1/2000 in assay buffer. The plate was washed and then incubated at room temperature for one hour with 50 μl/well of Extra-Avidin Alkaline Phosphatase (EA, Sigma), diluted 1/1000 in assay buffer. The plate was washed and 50 μl/well substrate buffer (2 mg/ml pNPP/10% diethanolamine/0.5mM MgCl_2_) was added to the wells. The plate was incubated in the dark for 20 minutes, and then production of the yellow chromophore at 405 nm was measured every ten minutes using the Synergy Neo2 (BioTek, USA) plate reader.

*CM-sepharose extraction fMPO activity assay:*

Faecal samples were solubilized 1/100 (v/v) in CTAB faecal solubilization buffer as above. Faecal samples were diluted at least 1/5 in CTAB faecal solubilization buffer before being added to the assay. For wash steps, the CM-sepharose beads were inverted at least 10 times, centrifuged at 8,6000 x g at room temperature for one minute, and the supernatant was then aspirated. 50 μl of CM-sepharose beads (GE Healthcare, Sweden) were equilibrated with 300 μl of 10 mM phosphate buffer (pH 7), and the supernatant was removed. 100 μl of solubilized faecal sample and 100 μl of 10 mM phosphate buffer (pH 7) were added to 50 μl of CM-sepharose beads. The samples were incubated on the beads with rotation for at least 30 minutes at room temperature. The beads were centrifuged, and the supernatant was removed. The CM-sepharose beads were then washed with 200 μl of 0.1% (v/v) Tween20/10 mM phosphate buffer (pH 7), 200 μl of 100% acetonitrile, and three subsequent washes of 200 μl of 10 mM phosphate buffer (pH 7). The supernatant was removed before addition of 10 μl reaction mixture (final concentration in 60 μl beads/buffer: 10 mM sodium nitrite/20 μM H_2_O_2_/50 μM AmplexUltraRed in 10 mM phosphate buffer (pH 7)). The beads were agitated for two minutes, at which point catalase (40 μg/μl) was added to the beads to stop the reaction. The resulting fluorophore was eluted from the CM-sepharose beads by addition of 200 μl of acetonitrile. The beads were centrifuged at 8,600 x g, and the supernatant was then added to a 96 well plate to allow reading of the fluorescent product at excitation wavelength of 544 nm and an emission wavelength at 590 nm, using a Synergy Neo2 (BioTek) fitted with a 530/590 compatible cube.

## Supplementary Figures


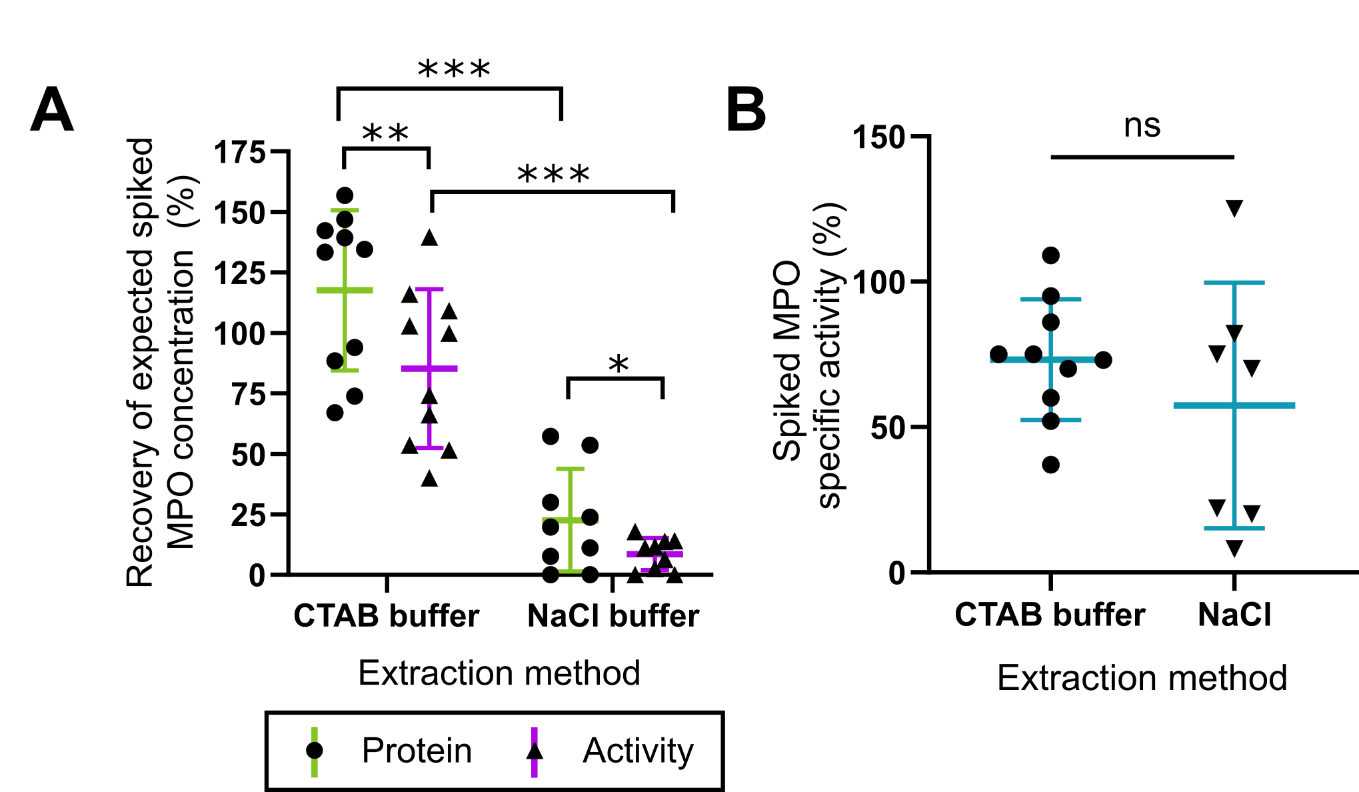


Supplementary Figure 1 Improved extraction of myeloperoxidase from faecal samples with CTAB solubilization buffer

Whole faecal samples (10 mg) from 10 patient samples were spiked with either buffer alone or 100 ng/μl purified human peroxidase, then extracted either into CTAB solubilization buffer (0.2% CTAB/0.00025% Tween20/0.1% BSA in PBS pH 7.4) or into NaCl buffer (500 mM NaCl in PBS pH 7.4). Accounting for dilutions, this led to an expected spiking of 10 ng/ml myeloperoxidase per well. Myeloperoxidase measured in the unspiked sample was subtracted from the myeloperoxidase measured in spiked samples. (**A**) Recovery of spiked myeloperoxidase from whole faeces using the CTAB solubilization buffer or NaCl buffer. Both protein and activity were measured for each sample. (**B**) Specific activity of myeloperoxidase recovered from spiked faecal samples. Abbreviations used; MPO, myeloperoxidase. Error bars represent mean ± SD Student’s t-test conducted using GraphPad Prism. ns = *P* > .05, **P* < .05, ***P* < .01, *****P* < .0001

**
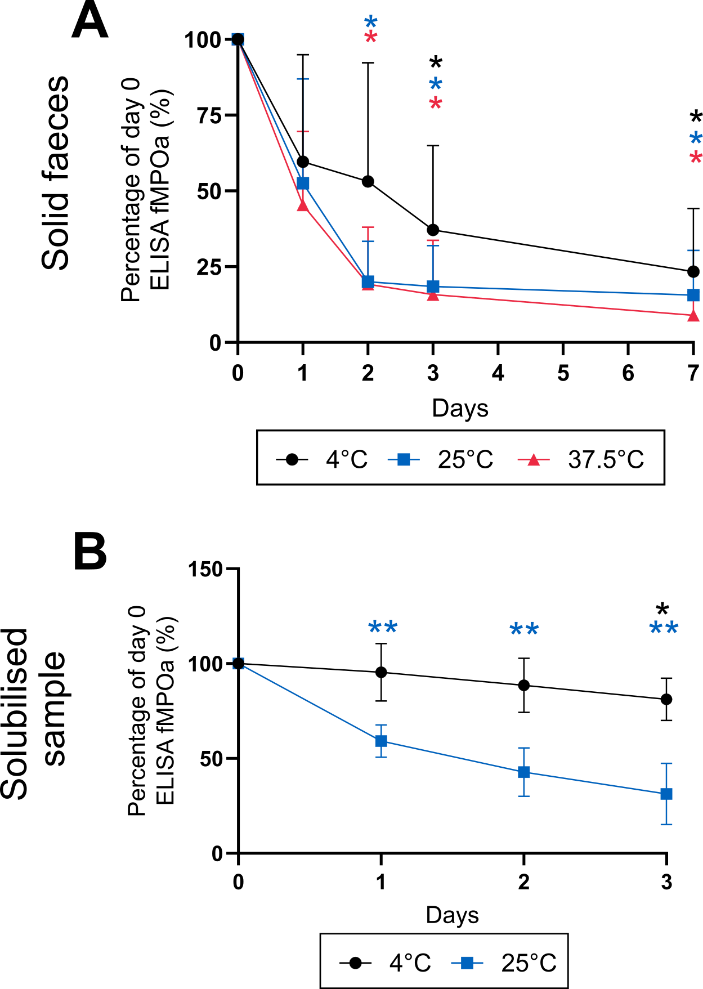
**

Supplementary Figure 2 Thermal stability of myeloperoxidase activity in faecal samples

(**A**) Stability of fMPO activity in whole faeces at different storage conditions. Six whole faeces samples were incubated at 4, 25 or 37.5°C for up to seven days. fMPO activity was measured by ELISA at each time point and was compared to measured fMPO at baseline (day 0). (**B**) Stability of fMPO activity in CTAB solubilization buffer in different storage conditions. Faecal samples in CTAB solubilization buffer were incubated at 4 or 25°C for up to three days. fMPO activity was measured by ELISA at each time point and was compared to measured fMPO at baseline (day 0). One-way ANOVA was used to examine each temperature condition, with planned multiple comparisons to day 0, and was conducted in GraphPad Prism. The graphed dots represent the mean, with coloured bars indicating ± SD. Abbreviations used; fMPO, faecal myeloperoxidase; MPO, myeloperoxidase. Data of equivalent experiments performed on fMPO protein thermal stability was previously published as supplementary material.^3^ **P* < .05, ***P* < .01


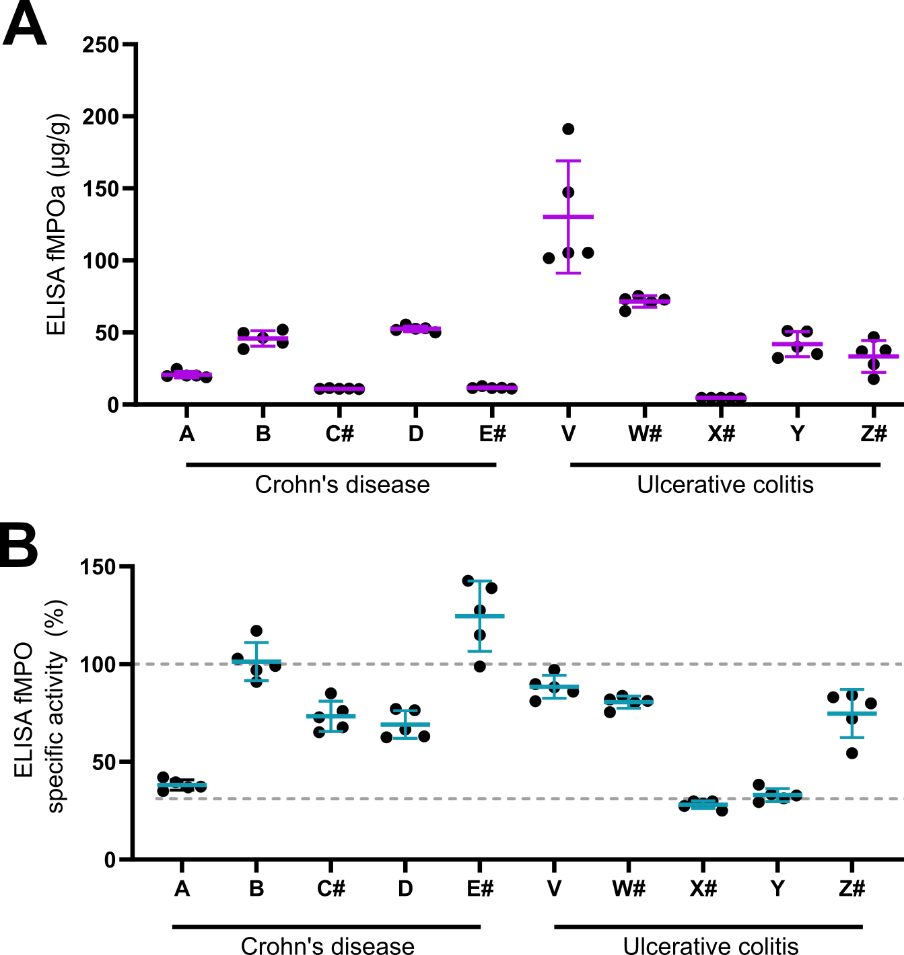


Supplementary Figure 3 Intra-sample variability of faecal myeloperoxidase in IBD samples

Ten whole faecal samples (five Crohn’s disease, five ulcerative colitis) were solubilized five times using RIDA faeces extraction devices filled with CTAB solubilization buffer. Samples that were semi-solid are marked with a “#” symbol. Each solubilized faecal sample was added to the fMPO ELISA, and (**A**) fMPO activity was measured. (**B**) Specific activity was calculated by dividing the fMPO activity concentration by the fMPO protein concentration for each sample. Abbreviations used: fMPO, faecal myeloperoxidase. Data of corresponding experiments performed on fMPO protein intra-sample variability has previously been published as supplementary material.^3^


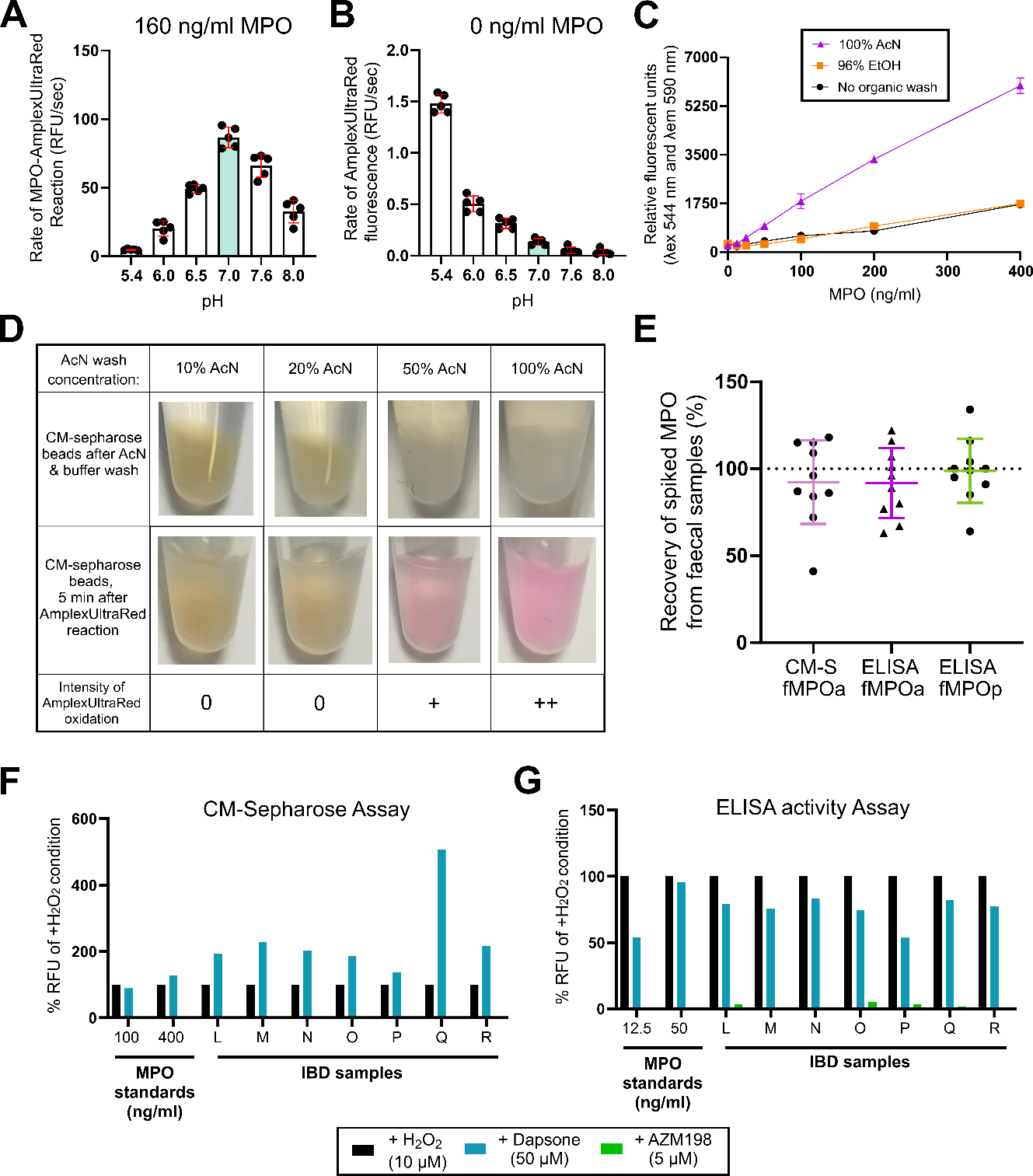


**Supplementary Figure 4 Development steps of the CM-Sepharose faecal myeloperoxidase extraction assay**

The oxidation of AmplexUltraRed by (**A**) 160 ng/ml myeloperoxidase or (**B**) no myeloperoxidase in 100 mM phosphate buffer at pH 5.4, 6, 6.5, 7, 7.6 or 8, added to a 96 well plate, was measured every 30 seconds for 25 minutes. The initial linear rate of AmplexUltraRed oxidation was calculated for each pH condition. The reaction mixture contained 10 mM sodium nitrite/50 μM AmplexUltraRed/20 μM H_2_O_2._ (n=5) for these experiments, mean and SD are graphed. (**C**) Myeloperoxidase was purified by the CM-sepharose extraction assay, and washed with either no organic solvent wash, 96% ethanol, or 100% acetonitrile. 10 mM sodium nitrite/50 μM AmplexUltraRed/20 μM H_2_O_2_ in 10 mM phosphate buffer (pH 7) was added to the beads and left to react for two minutes before the reaction was stopped by addition of 40 μg/ml catalase. The resultant fluorophore was eluted by 100% acetonitrile for reading on a 96-well plate. Shown is an example standard curve taken from three experimental repeats. (**D**) Photographs of CM-sepharose beads after incubation a solubilized faecal sample which had documented interference problems with the resultant activity assay. Photographs are taken before and after being washed with either 10, 20, 50 or 100% (v/v) acetonitrile diluted in 100 mM phosphate buffer (pH 7), and then subsequent reaction with AmplexUltraRed for 5 minutes. (**E**) Myeloperoxidase was spiked into either CTAB solubilization buffer (n=5), or into faecal samples in CTAB solubilization buffer with no detectable faecal myeloperoxidase by previous ELISA (n=10). Samples were diluted 1/5 in CTAB solubilization buffer before being added to the CM-sepharose assay, or 1/10 before being added to the fMPO ELISA. Signal from unspiked faecal samples were subtracted from the spiked samples before analysis. Recovery was calculated as percent recovery compared to spiked buffer samples. (**F-G**) Faecal samples were processed either by the (**F**) CM-sepharose fMPO extraction assay or the (**G**) ELISA fMPO activity assay. Prior to addition of the enzyme reaction mixture, either 10 μM H_2_O_2_ (H_2_O_2_ control), 50 μM Dapsone/10 μM H_2_O_2_ or 5 μM AZM198/10 μM H_2_O_2_ was added to each condition and incubated for 20 minutes at room temperature. The enzyme reaction mixture was then added to each assay; (**F**) 10 mM sodium nitrite/50 μM AmplexUltraRed/20 μM H_2_O_2_ in 10 mM phosphate buffer (pH 7), or (**G**) 50 mM NaBr/20 μM H_2_O_2_/50 μM AmplexUltraRed in 50 mM phosphate buffer (pH 7.4). The resultant AmplexUltraRed oxidation fluorescence for each condition was then compared to the fluorescence of the H_2_O_2_ control condition. (**F-G**) The signal for the AZM198 treated samples has been plotted on both graphs, though the signal was undetectable in all samples in (**F**).


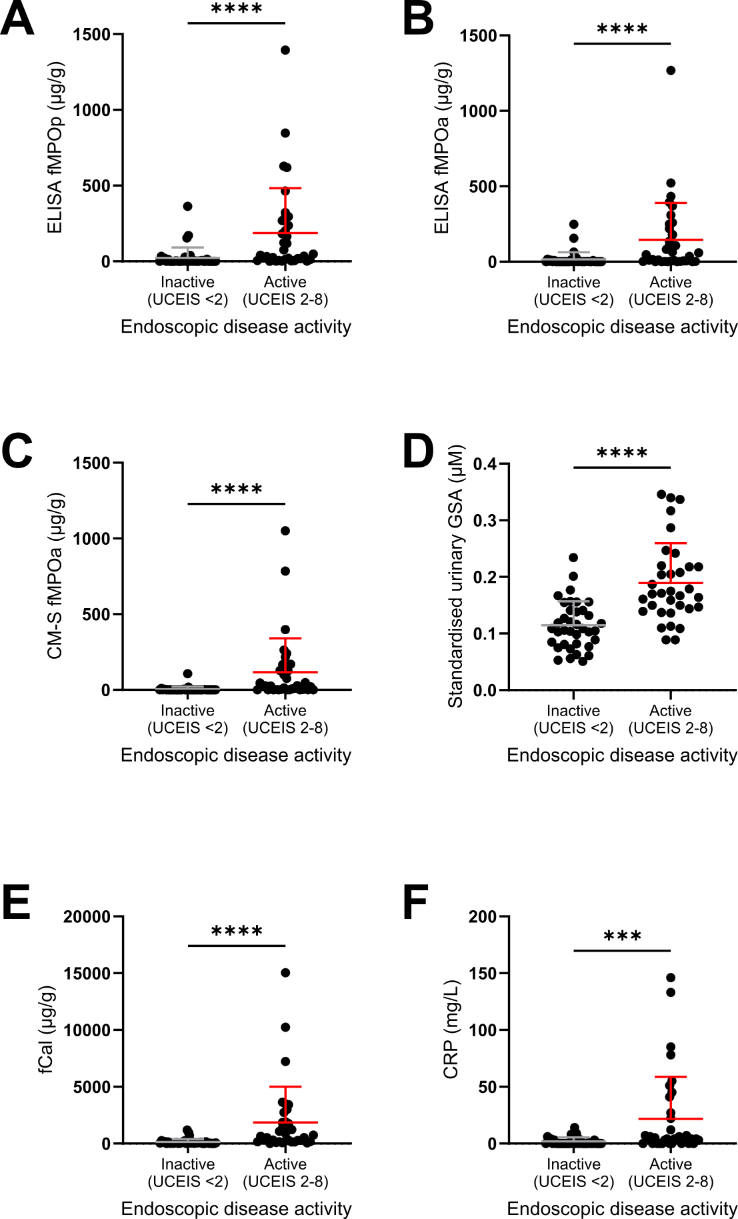


**Supplementary Figure 5 Performance of investigated biomarkers in ulcerative colitis stratified into inactive or active endoscopic activity**

Ulcerative colitis patients (n=72) were stratified into endoscopically inactive (UCEIS < 2, n=37) or active (UCEIS 2-8, n=35) disease activity. The following biomarkers were compared: (**A**) ELISA fMPOp, (**B**) ELISA fMPOa, (**C**) CM-S fMPOa and (**D**) Standardised urinary GSA. Data were analysed using GraphPad Prism to conduct Mann-Whitney U test. Grey and red bars represent mean ± SD. ****P*<.001, **** *P<*.0001.


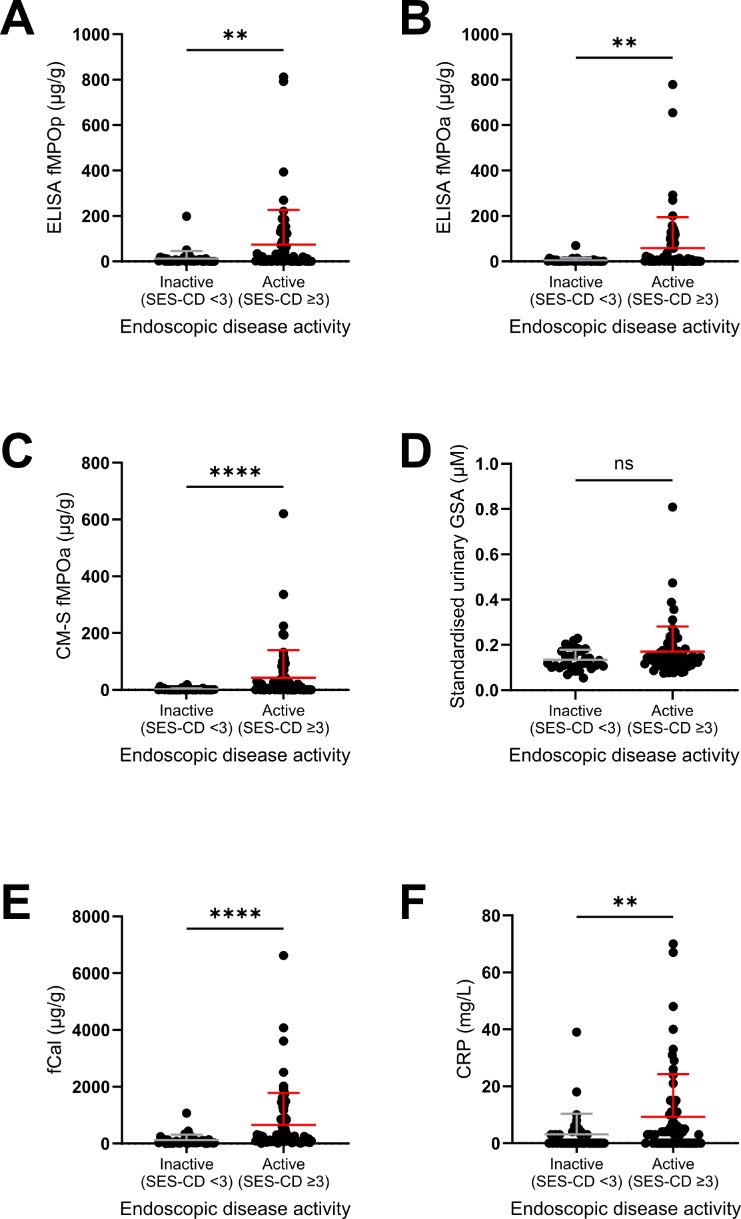


**Supplementary Figure 6 Performance of investigated biomarkers in Crohn’s disease stratified into inactive or active endoscopic activity**

Crohn’s disease patients (n=100) were stratified into endoscopically inactive (SES-CD < 3, n=36) or active (SES-CD ≥ 3, n=64) endoscopic disease activity. The following biomarkers were compared: (**A**) ELISA fMPOp, (**B**) ELISA fMPOa, (**C**) CM-S fMPOa and (**D**) Standardised urinary GSA. Data were analysed using GraphPad Prism to conduct Mann-Whitney U test. Grey and red bars represent mean ± SD. ***P<.*01, **** *P<*.0001.


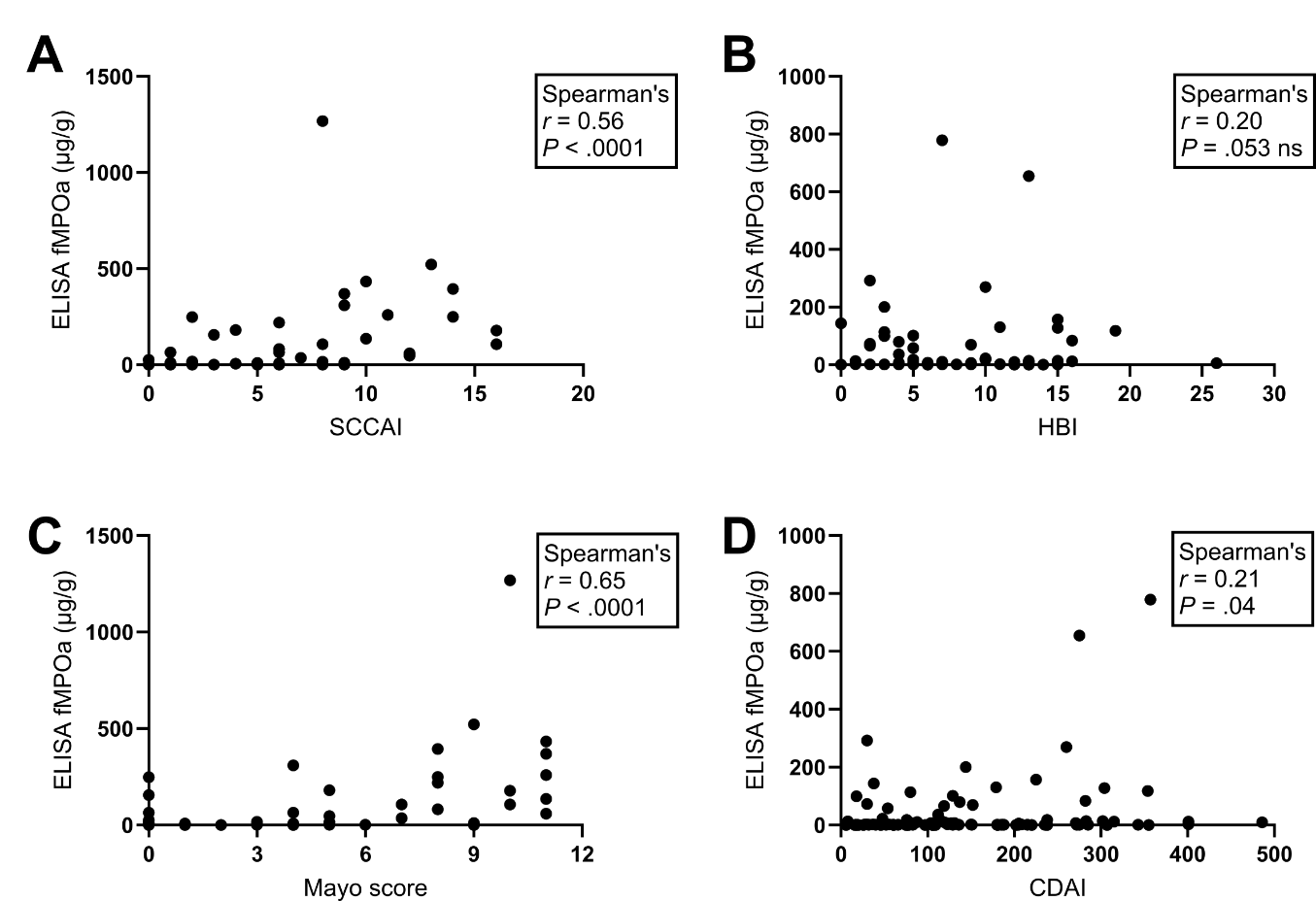


Supplementary Figure 7 Correlation of ELISA fMPOa and other clinical scores of IBD activity

Correlations between ELISA measured faecal myeloperoxidase activity (ELISA fMPOa) and clinical scores of disease activity in inflammatory bowel disease (IBD): (**A**) Simple clinical colitis activity index (SCCAI) (n=72), (**B**) Harvey-Bradshaw Index (HBI) (n=100), (**C**) Mayo Score (n=72) and (**D**) Crohn’s disease activity index (CDAI) (n=100). Spearman’s correlations were conducted in GraphPad Prism; *r* and *P* values are shown on each graph.
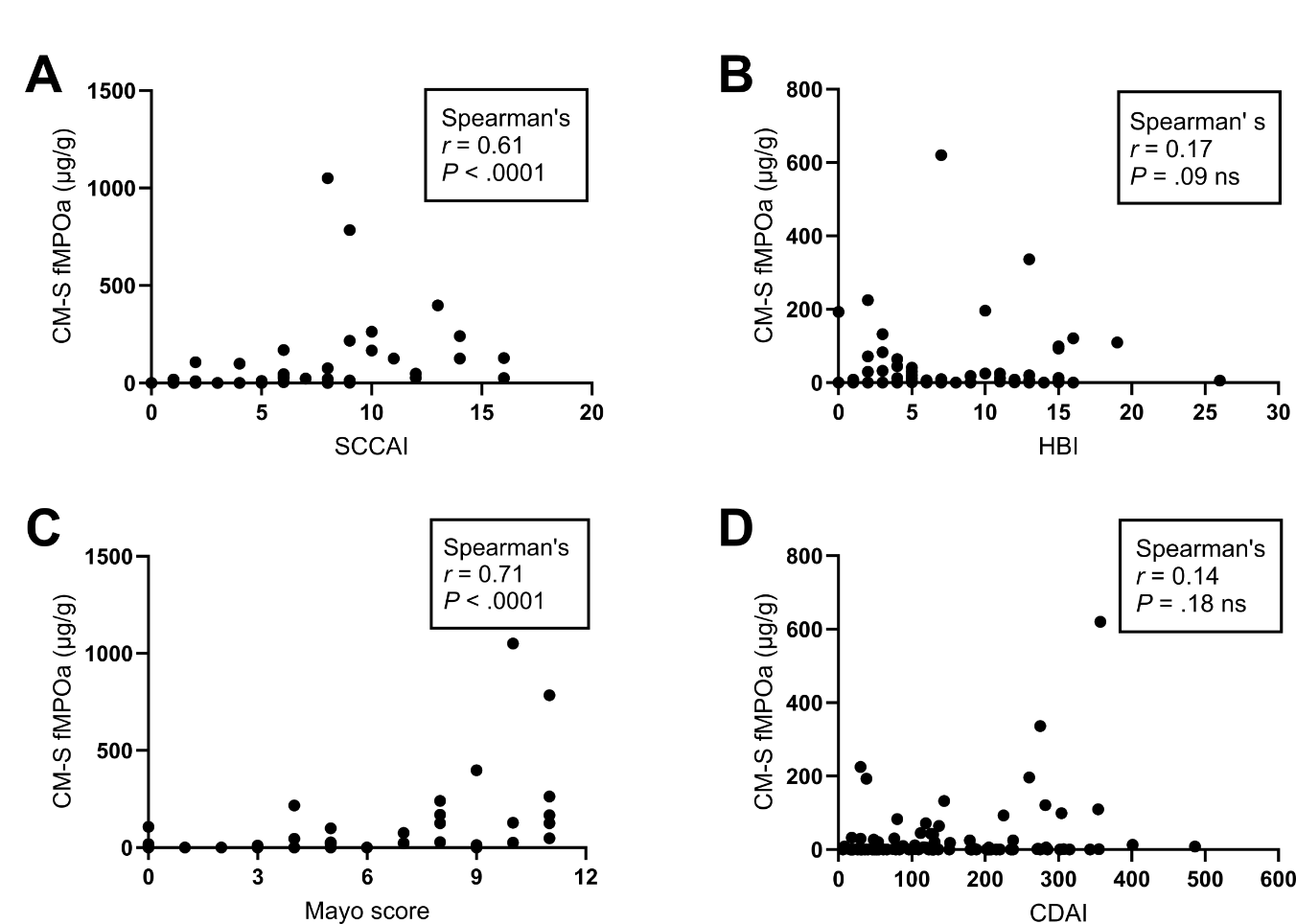


**Supplementary Figure 8** **Correlation of CM-S fMPOa to other clinical scores of IBD activity**

Correlations between CM-Sepharose extraction assay measured faecal myeloperoxidase activity (CM-S fMPOa) and clinical scores of disease activity in inflammatory bowel disease (IBD): (**A**) Simple clinical colitis activity index (SCCAI) (n=72), (**B**) Harvey-Bradshaw Index (HBI) (n=100), (**C**) Mayo Score (n=72) and (**D**) Crohn’s disease activity index (CDAI) (n=100). Spearman’s correlations were conducted in GraphPad Prism; *r* and *P* values are shown on each graph.


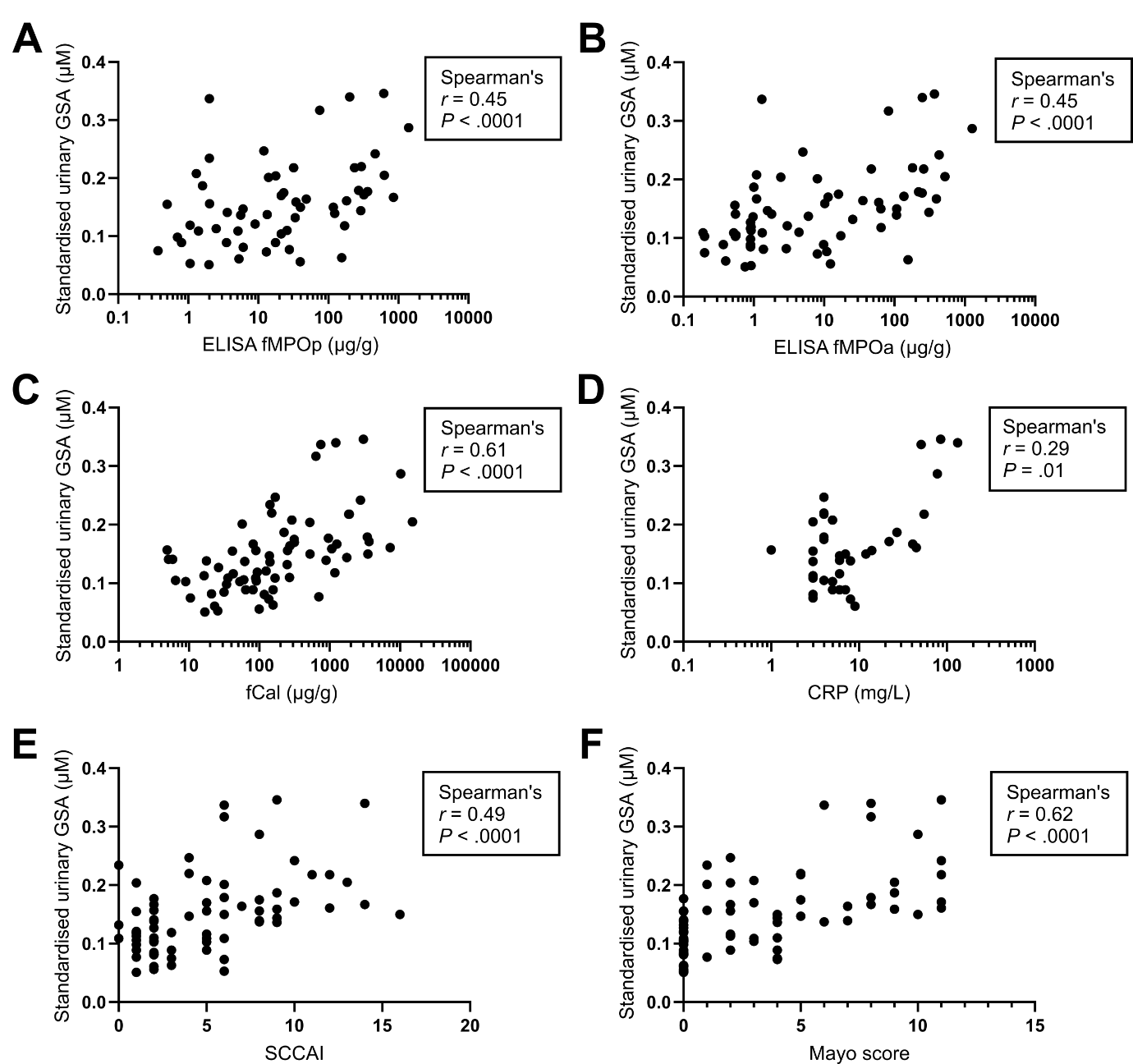


Supplementary Figure 9 Correlations of urinary GSA and biomarkers or clinical scores of ulcerative colitis

Correlations between standardized urinary glutathione sulfonamide (GSA) and biomarkers or clinical scores of disease activity in ulcerative colitis (n = 71). (**A**) ELISA fMPOp, (**B**) ELISA fMPOa, (**C**) fCal, (**D**) CRP, (**E**) Simple clinical colitis activity index (SCCAI) and (**F**) Mayo score. The x axis on graphs A-D has been scaled to log_10_ for added clarity. Spearman’s correlations were conducted on non-logarithm data in GraphPad Prism; *r* and *P* values are shown on each graph.


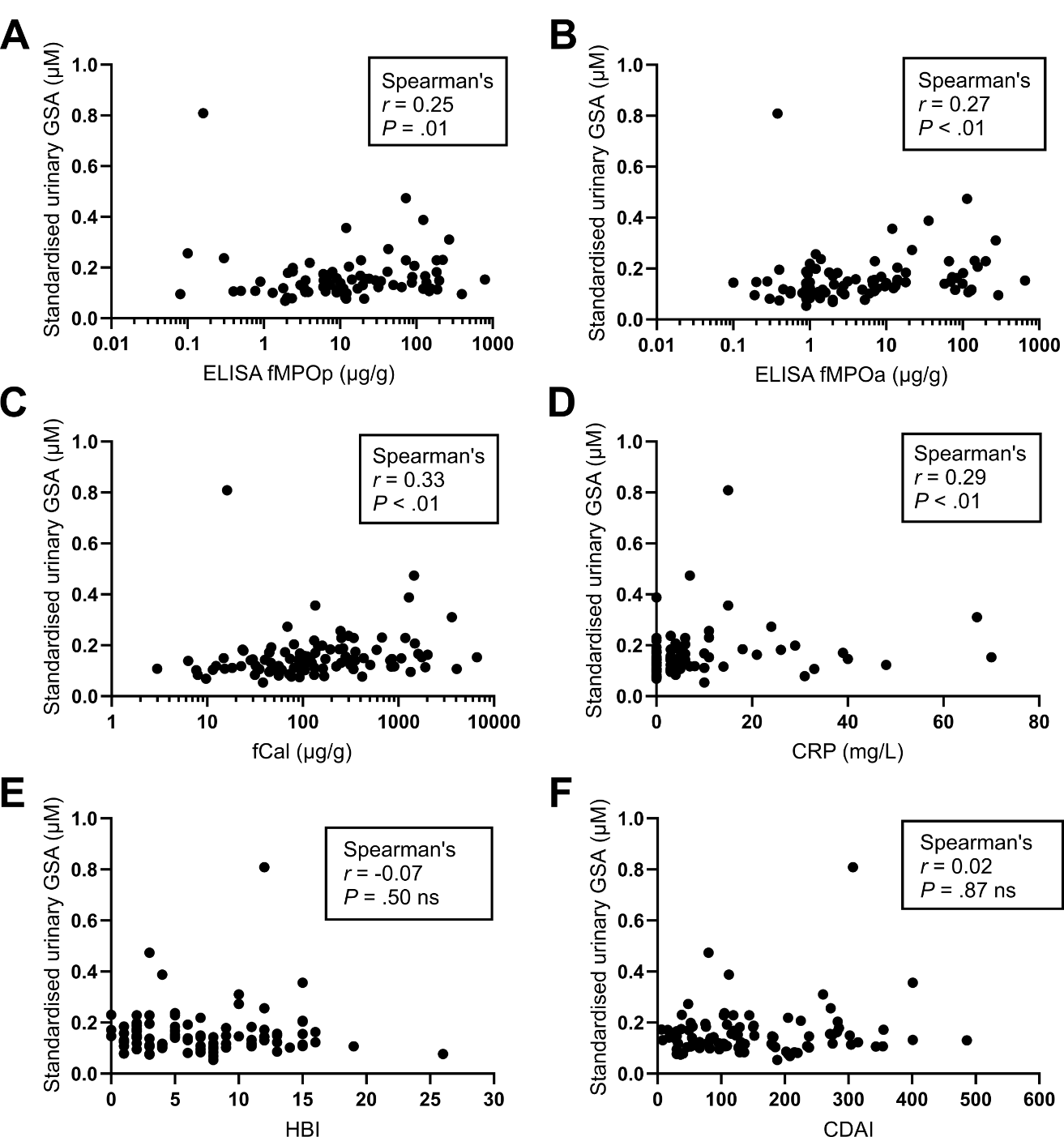


Supplementary Figure 10 Correlations of urinary GSA and biomarkers or clinical scores of Crohn’s disease.

Correlations between urinary GSA and biomarkers or clinical scores of IBD activity in Crohn’s disease (n=99). (**A**) ELISA fMPOp, (**B**) ELISA fMPOa, (**C**) fCal, (**D**) CRP, (**E**) Harvey-Bradshaw Index (HBI) and (**F**) Crohn’s disease activity index (CDAI). The x axis in graphs **A-C** have been presented on a log_10_ scale to better show the correlations. Spearman’s correlations were conducted on non-logarithm data in GraphPad Prism; *r* and *P* values are shown on each graph.


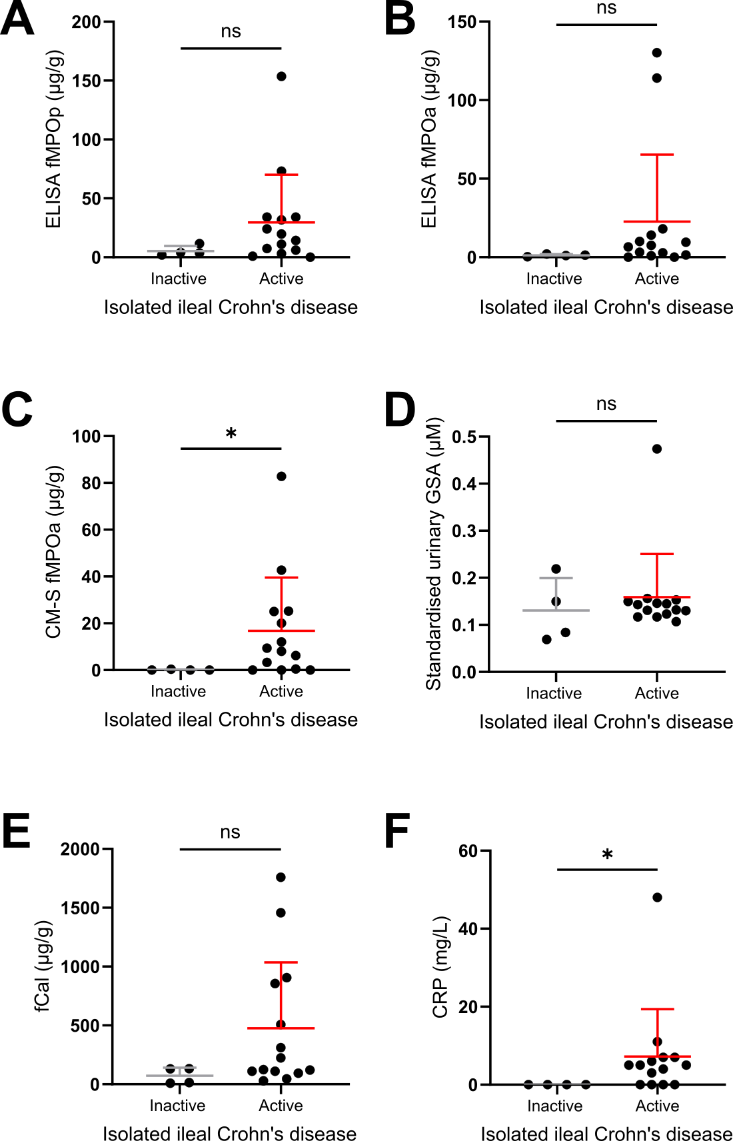


Supplementary Figure 11 Performance of investigated biomarkers in isolated ileal Crohn’s disease patients

Subgroup analysis of (**A**) ELISA fMPOp, (**B**) ELISA fMPOa, (**C**), CM-S fMPOa, (**D**) Standardised urinary GSA, (**E**) fCal, and (**F**) CRP in individuals with isolated ileal (Montreal location classification L1) Crohn’s disease (inactive n=4, active n=14). Data for fCal and CRP were analysed and published previously.^3^ Data were analysed using GraphPad Prism to conduct Mann-Whitney U test. Grey and red bars represent mean ± SD. **P<.*05.

## Supplementary Tables

Supplementary Table 1 Grouping of colonoscopic disease activity of NIDA-IBD cohort

|  | **IBD endoscopic activity scores** | |
| --- | --- | --- |
| **Grouped disease activity:** | **UCEIS** | **SES-CD** |
| **Inactive/Remission** | 0-1 | 0-2 |
| **Mild** | 2-4 | 3-6 |
| **Moderate** | 5-6 | 7-15 |
| **Severe** | 7-8 | 16 + |

Abbreviations used: SES-CD, simple endoscopic score for Crohn’s disease; UCEIS, ulcerative colitis endoscopic index of severity.

Supplementary Table 2 Descriptive statistics

|  | **UC (n = 72)** | **CD (n = 100)** | **HC:**  **faecal (n=10);**  **urine (n=10)** |
| --- | --- | --- | --- |
| **#Endoscopic remission [%]** SES-CD ≤ 2, UCEIS < 2 | 37 [51.4] | 38 [38] | N/A |
| **#Endoscopically active disease [%]** SES-CD > 2, UCEIS ≥ 2 | 35 [48.6] | 62 [62] | N/A |
| **#Mild endoscopic activity [%]** | 22 [30.6] | 34 [34] | N/A |
| **#Moderate-severe endoscopic activity [%]** | 13 [18] | 28 [28] | N/A |
| **#Median C-reactive protein**  (mg/L) **[IQR]** | 3 [0-6] | 3  [0-6.75] | N/A |
| **#Median faecal calprotectin**  (μg/g) **[IQR]** | 146.1  [45.2-853.1] | 115.6  [45.84-350.3] | 27.9  [24.4-64.18] |
| **Median ELISA measured fMPO enzyme activity**  (μg/g) **[IQR]** | 3 [0.9-64.5] | 2 [0.9-14] | 1.6 [1.5-1.7] |
| **Median ELISA measured fMPO protein concentration**  (μg/g) **[IQR]** | 10.5 [0.9-107.3] | 7.05  [0.3-34.12] | 0.06 [0-1.1] |
| **Median CM-sepharose extraction assay measured fMPO enzyme activity**  (μg/g) **[IQR]** | 0.6  [0-26.6] | 0.61 [0-16.95] | 0 [0-0.5] |
| **Median standardized urinary GSA** (μM) **[IQR]** | 0.141  [0.104-0.177] | 0.14  [0.108-0.175] | 0.14  [0.098-0.16] |
| **Median urine specific gravity**  **[IQR]** | 1.018  [0.011-1.024] | 1.017  [1.011-1.042] | 1.013  [1.008-1.019] |

Abbreviations used: CD, Crohn’s disease; fMPO, faecal myeloperoxidase; GSA, glutathione sulfonamide; HC, healthy controls; IQR, interquartile range; SES-CD, simple endoscopic score for Crohn’s disease; UCEIS, ulcerative colitis endoscopic index of severity. # Data were published previously.^3^

Supplementary Table 3 Spearman's correlations of measures of fMPO activity with IBD clinical scores in patients with endoscopically active disease

|  | **IBD clinical scores** | | | | | |
| --- | --- | --- | --- | --- | --- | --- |
| **Biomarkers** | **UCEIS**  (n = 35) | **Mayo**  (n = 35) | **SCCAI**  (n = 35) | **SES-CD**  (n = 62) | **CDAI**  (n = 62) | **HBI**  (n = 62) |
| **ELISA fMPOa** | 0.68* | 0.67* | 0.61* | 0.60* | 0.23 | 0.20 |
| **CM-S fMPOa** | 0.67* | 0.67* | 0.64* | 0.54* | 0.18 | 0.23 |
| **Urinary GSA** | 0.34  (p = 0.05) | 0.42* | 0.27 | 0.27* | -0.03 | -0.07 |

* = statistically significant (*P*<.05). Abbreviations used: CDAI, Crohn’s disease activity index; CM-S fMPOa, CM-sepharose extraction assay measured faecal myeloperoxidase activity; ELISA fMPOa, ELISA-measured faecal myeloperoxidase activity; HBI, Harvey-Bradshaw index; SCCAI, simple clinical colitis activity index; SES-CD, simple endoscopic score for Crohn’s disease; UCEIS, ulcerative colitis endoscopic index of severity.

Supplementary Table 4 Combination biomarkers AUROC

| **Disease** | **Disease grouping** | **Best solo biomarker (AUROC)** | **Combination** | **AUROC** | **Sensitivity (%)** | **Specificity (%)** | **p value (paired sample area AUROC difference)** |
| --- | --- | --- | --- | --- | --- | --- | --- |
| **Crohn’s disease** | **remission-mild vs moderate-severe** | **fCal (0.86)** | fCal + fMPOa | 0.88 | 85 | 74 | 0.16 |
|  |  |  | fCal + fMPOp | 0.87 | 67 | 93 | 0.37 |
|  |  |  | fCal + uGSA | 0.87 | 82 | 81 | 0.75 |
|  |  |  | fMPOa + uGSA | 0.87 | 85 | 78 | 0.70 |
|  |  |  | fMPOp + uGSA | 0.87 | 85 | 80 | 0.87 |
|  |  |  | CM-S fMPOa + fCal | 0.88 | 63 | 100 | 0.43 |
|  |  |  | CM-S fMPOa + ELISA fMPOa | 0.87 | 63 | 100 | 0.83 |
|  |  |  | CM-S fMPOa + ELISA fMPOp | 0.87 | 63 | 99 | 0.79 |
|  | **inactive vs active** | **fCal (0.76)** | fCal + CRP | 0.77 | 67 | 81 | 0.81 |
|  |  |  | fCal + ELISA fMPOa | 0.77 | 87 | 56 | 0.85 |
|  |  |  | fCal + CM-S fMPOa | 0.77 | 64 | 81 | 0.86 |
|  |  |  | fCal + HBI | 0.78 | 90 | 54 | 0.59 |
|  |  |  | ELISA fMPOa + CM-S fMPOa | 0.77 | 57 | 86 | 0.90 |
| **Ulcerative colitis** | **remission-mild vs moderate-severe** | **fCal (0.94)** | ELISA fMPOa + SCCAI | 0.94 | 100 | 83 | 0.69 |
|  |  |  | ELISA fMPOp + SCCAI | 0.95 | 100 | 85 | 0.37 |
|  |  |  | CM-S fMPOa | 0.94 | 100 | 86 | 0.75 |
|  |  |  | CM-S fMPOa + fCal | 0.94 | 100 | 86 | 0.75 |
|  |  |  | CM-S fMPOa + ELISA fMPOa | 0.94 | 100 | 85 | 0.74 |
|  |  |  | CM-S fMPOa + SCCAI | 0.94 | 100 | 85 | 0.54 |
|  | **inactive vs active** | **fCal (0.88)** | fCal + CM-S fMPOa | 0.89 | 86 | 81 | 0.19 |
|  |  |  | uGSA + SCCAI | 0.9 | 79 | 95 | 0.63 |

Abbreviations used: CM-S fMPOa, CM-Sepharose measured faecal myeloperoxidase activity; CRP, C-reactive protein; ELISA fMPOa, ELISA-measured faecal myeloperoxidase activity; ELISA fMPOp, ELISA-measured faecal myeloperoxidase protein; fCal, faecal calprotectin; HBI, Harvey-Bradshaw index; SCCAI, simple clinical colitis activity index; uGSA, urinary glutathione sulfonamide. The data was reanalysed for this publication, but data for fCal and CRP were published previously.^3^

Supplementary Table 5 – Isolated ileal Crohn’s disease subgroup analysis

|  | **All ileal CD patients** (n = 18) | **Active ileal CD**  (n = 14) | **Inactive ileal CD**  (n = 4) | **Mann-Whitney U test**  (p values) |
| --- | --- | --- | --- | --- |
| **Median C-reactive protein** (mg/L) **[IQR]** | 3.5 [0-6.25] | 5 [0-7] | 0 [0-0] | 0.037* |
| **Median faecal calprotectin** (μg/g) **[IQR]** | 128 [83-594] | 174 [107-868] | 73 [11-132] | 0.158 |
| **Median ELISA-measured fMPO activity** (μg/g) **[IQR]** | 3.0 [0.9-11] | 7.1 [1.3-15] | 1.2 [0-1.8] | 0.101 |
| **Median ELISA-measured fMPO protein** (μg/g) **[IQR]** | 11.3 [3.4-32.4] | 17 [5.3-34] | 3.8 [2.3-9.7] | 0.158 |
| **Median CM-Sepharose-measured fMPO activity** (μg/g) **[IQR]** | 4.7 [0-21.3] | 8.7 [0.3-25.1] | 0 [0-0.3] | 0.024* |
| **Median standardized urinary GSA** (μM) **[IQR]** | 0.138 [0.117-0.1508] | 0.138 [0.122-0.151] | 0.117 [0.073-0.202] | 0.664 |

* = statistically significant (*P*<.05). Abbreviations used: CD, Crohn’s disease; fMPO, faecal myeloperoxidase; GSA, glutathione sulfonamide; IQR, interquartile range. Data for fCal and CRP were published previously.^3^

## Supplementary References

1. Odajima T, Yamazaki I. Myeloperoxidase of the leukocyte of normal blood. I. Reaction of myeloperoxidase with hydrogen peroxide. Biochimica et Biophysica Acta (BBA) - Enzymology. 1970;206(1):71-77.

2. Hoskin T. Neutrophil Activation in Inflammatory Bowel Disease. 2011.

3. Swaminathan A, Borichevsky GM, Edwards TS, et al. Faecal Myeloperoxidase as a Biomarker of Endoscopic Activity in Inflammatory Bowel Disease. Journal of Crohn’s and Colitis. July 2022:jjac098.
